# Biosynthesis of Antibiotic Leucinostatins in Bio-control Fungus *Purpureocillium lilacinum* and Their Inhibition on *Phytophthora* Revealed by Genome Mining

**DOI:** 10.1371/journal.ppat.1005685

**Published:** 2016-07-14

**Authors:** Gang Wang, Zhiguo Liu, Runmao Lin, Erfeng Li, Zhenchuan Mao, Jian Ling, Yuhong Yang, Wen-Bing Yin, Bingyan Xie

**Affiliations:** 1 Institute of Vegetables and Flowers, Chinese Academy of Agricultural Sciences, Beijing, PR China; 2 State Key Laboratory of Mycology, Institute of Microbiology, Chinese Academy of Sciences, Beijing, PR China; 3 College of Life Sciences, Beijing Normal University, Beijing, PR China; Texas A&M University, UNITED STATES

## Abstract

*Purpureocillium lilacinum* of Ophiocordycipitaceae is one of the most promising and commercialized agents for controlling plant parasitic nematodes, as well as other insects and plant pathogens. However, how the fungus functions at the molecular level remains unknown. Here, we sequenced two isolates (PLBJ-1 and PLFJ-1) of *P*. *lilacinum* from different places Beijing and Fujian. Genomic analysis showed high synteny of the two isolates, and the phylogenetic analysis indicated they were most related to the insect pathogen *Tolypocladium inflatum*. A comparison with other species revealed that this fungus was enriched in carbohydrate-active enzymes (CAZymes), proteases and pathogenesis related genes. Whole genome search revealed a rich repertoire of secondary metabolites (SMs) encoding genes. The non-ribosomal peptide synthetase LcsA, which is comprised of ten C-A-PCP modules, was identified as the core biosynthetic gene of lipopeptide leucinostatins, which was specific to *P*. *lilacinum* and *T*. *ophioglossoides*, as confirmed by phylogenetic analysis. Furthermore, gene expression level was analyzed when PLBJ-1 was grown in leucinostatin-inducing and non-inducing medium, and 20 genes involved in the biosynthesis of leucionostatins were identified. Disruption mutants allowed us to propose a putative biosynthetic pathway of leucinostatin A. Moreover, overexpression of the transcription factor *lcsF* increased the production (1.5-fold) of leucinostatins A and B compared to wild type. Bioassays explored a new bioactivity of leucinostatins and *P*. *lilacinum*: inhibiting the growth of *Phytophthora infestans* and *P*. *capsici*. These results contribute to our understanding of the biosynthetic mechanism of leucinostatins and may allow us to utilize *P*. *lilacinum* better as bio-control agent.

## Author Summary

*Purpureocillium lilacinum*, a well-known bio-control agent against various plant pathogens in agriculture, can produce antibiotic leucinostatins—peptaibiotic with extensive biological activities, including antimalarial, antiviral, antibacterial, antifungal, and antitumor activities, as well as phytotoxic. We have sequenced the genomes of two *P*. *lilacinum* isolates, and compared them with other fungi, focusing on their bio-control characteristics. We discovered a rich repertoire of CAZymes, proteases, SMs and pathogenesis related genes. We also identified a gene cluster containing 20 genes involved in the leucinostatins A and B biosynthesis by gene deletion, qRT-PCR and RNA-seq analyses. A transcription factor in the pathway was overexpressed, resulting in the upregulation of the related genes and a 1.5-fold increase in leucinostatins A and B. A new bioactivity of leucinostatins, inhibition of the growth of the notorious *Phytophthora*, was identified in this study by confronting incubation with *P*. *lilacinum*. These results provided new strategies for the agricultural development of leucinostatins and improving *P*. *lilacinum* strains.

## Introduction

Plant parasitic nematodes with wide host ranges cause enormous crop and economic losses amounting to $157 billion annually worldwide [1, 2]. Biological control by fungi has become increasingly popular due to nematicides’ risks of environmental toxicity and adverse effects on human health [3]. One of the most promising and commercialized agents, *Purpureocillium lilacinum*, has been evaluated to assess its bio-control activity against plant nematodes in a number of studies [2, 4]. In particular, *P*. *lilacinum* has been reported to effectively control such species as the cotton aphid *Aphis gossypii* [5], the greenhouse whitefly *Trialeurodes vaporariorum*, the glasshouse red spider mite *Tetranychus urticae* [6], and the leaf-cutting ant *Acromyrmex lundii* [7].

**Fig 1:**
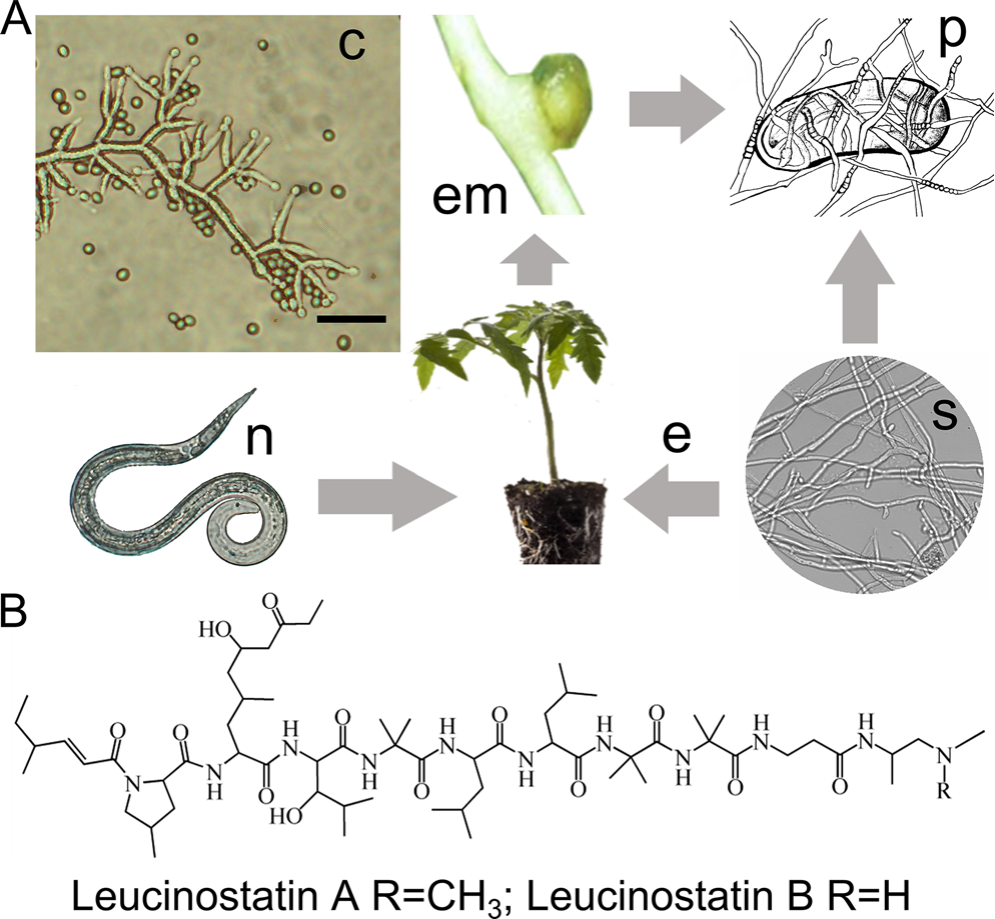
Lifestyles of nematophagous *P*. *lilacinum* and the structures of leucinostatins. (A) Microscopic conidiophores and conidia (c) of *P*. *lilacinum*. Scale bar = 10 μm. The soil saprophyte (s) *P*. *lilacinum* colonizes plant roots as an endophyte (e), and the parasite (p) can occur in nematode eggs in the egg mass (em) generated after the infection with the plant nematode (n). (B) Chemical structure of leucinostatins A and B.

The genus *Purpureocillium* was recently proposed for of Ophiocordycipitaceae, based on the internal transcribed spacer (ITS) and translation elongation factor 1-α (TEF) sequences of *P*. *lilacinum*, although it was originally classified in the genus *Paecilomyces* [8]. *P*. *lilacinum* is commonly isolated from soil, plant roots, nematodes and insects, and it occasionally infects people. This fungus employs flexible lifestyles, including soil-saprobes, plant-endophytes and nematode pathogens. Opportunistic infection occurs when nematode eggs encounter *P*. *lilacinum*; therefore, parasitism can be a mechanism for nematode bio-control (Fig 1A). It has now been confirmed that a serine protease [9], a cuticle-degrading protease [10] and chitinase [11] play important roles in infection by degrading nematode eggshells.

Recently, the production of SMs has been shown to be a mechanism that kills nematodes. For example, culture filtrates of *P*. *lilacinum*, in which leucinostatins were produced, caused strong mortality and inhibited nematode reproduction [12]. In addition to leucinostatins, a few other SMs have been isolated from *P*. *lilacinum*. The novel pyridone alkaloid paecilomide, an acetylcholinesterase inhibitor, was produced when this fungus was co-cultured with *Salmonella typhimurium* [13]. Two xanthone-anthraquinone heterodimers, acremoxanthone C and acremonidin A, were isolated in the course of a search for calmodulin ligands [14].

The leucinostatins (Fig 1B) are a family of lipopeptide antibiotics isolated from *P*. *lilacinum* [15], *Paecilomyces marquandii* [16–18] and *Acremonium sp*. [19]. Leucinostatin A contains nine amino acid residues, including the unusual amino acid 4-methyl-L-proline (MePro), 2-amino-6-hydroxy-4-methyl-8-oxodecanoic acid (AHyMeOA), hydroxyleucine (HyLeu), α-aminoisobutyric acid (AIB), β-Ala, and a 4-methylhex-2-enoic acid at the N-terminus as well as an N1,N1-dimethylpropane-1,2- diamine (DPD) at the C-terminus. Twenty-four different structures have been described in the leucinostatin series[20]. Leucinostatin A significantly suppressed prostate cancer growth in a coculture system in which prostate stromal cells stimulated the growth of DU-145 human prostate cancer cells through insulin-like growth factor I [21]. When screening for antitrypanosomal compounds among several peptide antibiotics, leucinostatins showed the most potent activity against trypanosomes. Trypanosome infection causes human African trypanosomiasis, which is one of the world’s most neglected diseases lacking satisfactory drugs [22]. Furthermore, leucinostatins have displayed broad bioactivity against bacteria and fungi. These antibiotics’ functions are based on their ability to inhibit ATP synthesis in the mitochondria as well as different phosphorylation pathways [23]. These findings drew our attention to the relationships between the bio-control function of *P*. *lilacinum* and leucinostatins. Furthermore, genetic and molecular information regarding the biosynthesis of this family of lipopeptide antibiotics, of which little was known to date, could contribute to increasing its production and screening for more efficient derivative compounds.

Genome sequences have shed light on the mechanism of the endoparasitic lifestyle or nematode control beyond biological research. During the preparation of our manuscript, the genome sequence of *P*. *lilacinum* was published [24]. Two other plant nematode endoparasitic fungi, *Pochonia chlamydosporia* [25] and *Hirsutella minnesotensis* [26], were recently sequenced. Genome sequencing revealed that *P*. *chlamydosporia* encoded a wide array of hydrolytic enzymes and transporters expressed at the mRNA level, which supported its multitrophic lifestyle, and *H*. *minnesotensis*, which mainly invades juvenile stage cyst nematodes, putatively conducted its parasitic process through lectins, secreted proteases and SMs. Thus, the genome sequence of *P*. *lilacinum* provides an opportunity to better understand its mechanism in controlling plant nematodes, and it would be useful to enhance its capabilities as a bio-control agent. At the same time, the genome sequence has the potential to solve the biosynthetic puzzle of leucinostatins as well as to detect novel genes and metabolites that might be of value in agriculture and medicine.

Here, we present the results of genome sequencing of the PLBJ-1 and PLFJ-1 strains of the bio-control agent *P*. *lilacinum*, and we increased our knowledge of its bio-control capabilities by comparing the sequences of *P*. *lilacinum* with those of other fungi. The genome revealed a repertoire of SM-encoding genes that illustrated the potential for using this fungus to discover natural products. Furthermore, we identified the leucinostatin gene cluster (*lcs* cluster) and proposed a hypothetical pathway for biosynthesis through genetic manipulation. In the course of screening for new activities of leucinostatins, we found that they inhibited the most notorious oomycetes *P*. *infestans*, which causes potato late blight and results in global yield losses of 16% [27].

## Results

### General structure of the *P*. *lilacinum* genome

**Fig 2:**
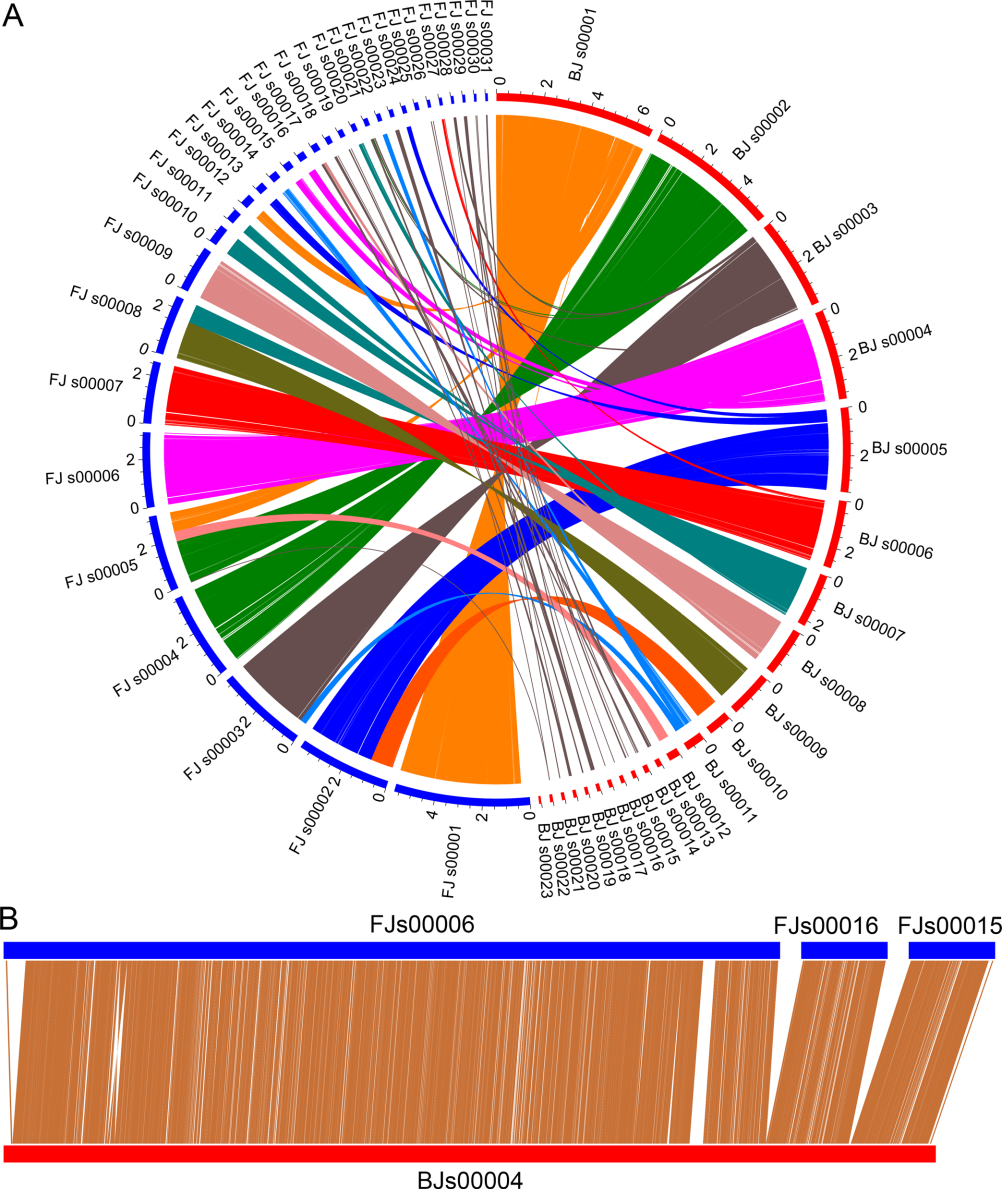
Genomic synteny of PLBJ-1 and PLFJ-1. (A) The syntenic genome sequences of PLBJ-1 and PLFJ-1 were analyzed by BLASTN, with an E-value cutoff of 1e-5. The red semicircle represents the scaffolds of PLBJ-1, while the blue semicircle represents the scaffolds of PLFJ-1. Scaffold lengths of ≥ 100 Kb were used for this analysis, and the threshold of matched blocks was ≥ 1000 bp, which are connected by lines of the same color. (B) An example of a super-scaffold inferred by syntenic analysis.

Two *P*. *lilacinum* isolates, PLBJ-1 and PLFJ-1, were sequenced to ensure the accuracy of the genome information and the subsequent analysis. PLBJ-1 and PLFJ-1 were assembled into 144 and 163 scaffolds, respectively, with total sizes of 38.14 and 38.53 Mb, while the published TERIBC I was assembled into 301 scaffolds with a total size of 38.82 Mb (Table 1). The comparative genome sizes of related fungi species are listed in S1 Table. A total of 11,773 and 11,763 gene models were predicted in both genomes, respectively, parallel to other ascomycetes fungi (S1 Table). BLASTN analysis was performed between the two genomes and demonstrated that 83.56% of the PLBJ-1 genome and 82.79% of the PLFJ-1 genome shared high synteny (Fig 2A). According to the syntenic relationship of PLBJ-1 and PLFJ-1, we reconstructed 10 super-scaffolds (S2 Table), which illustrated the physical ubieties of the assembled scaffolds; e.g., scaffold 00006, scaffold 00016 and scaffold 00015 in PLFJ-1 were combined into a super-scaffold (Fig 2B). The overall syntenic relationship of PLBJ-1 and TERIBC 1 showed that 76.52% of the PLBJ-1 genome and 75.12% of the TERIBC 1 genome shared high synteny (S1 Fig). Approximately 6.07% of the repeat sequences that included transposon elements (TEs) (~4.37%) and tandem repeats (~1.70%) were identified in PLBJ-1. The Class I (retrotransposons) and Class II (DNA transposons) TEs occupied ~1.80% and ~0.76% of the genome, respectively. The PLFJ-1 isolate harbored a similar number of repeat sequences (6.00%). The distribution of the TE families was similar in the two isolates, with the exception of certain families, e.g., I, Gypsy, Penelope, Tc1-Mariner and hAT (S3 Table). In total, the two isolates of *P*. *lilacinum* contained a larger number of retrotransposons than DNA transposons. *P*. *lilacinum* exhibited expansion of repeat content comparable to other ascomycetes fungi, with the exception of *H*. *minnesotensis*, *Ophiocordyceps sinensis* and *Fusarium oxysporum (fol)*, in which the repeat sequences accounted for more than one quarter of the genome (S1 Table). In TERIBC 1, approximately 1.68% of the genome sequence was identified as repeat content.

**Table 1:** Genome feature of the three *P*. *lilacinum* isolates.

**Feature**	**PLBJ-1**	**PLFJ-1**	**TERIBC 1**
Accession number	LSBH00000000	LSBI00000000	LOFA00000000.1
Length (Mb)	38.14	38.53	38.82
Scaffold number	144	163	301
Contig number	596	818	301
Max scaffold length (Mb)	6.69	5.70	3.7
Scaffold N50	3.77	3.20	1.79
G+C contents (%)	58.5	58.4	58.6
Repeat sequence (%)	6.07	6.00	1.68
Protein-coding genes	11,773	11,763	13,266
tRNA genes	90	90	91
Secreted proteins	1410	1448	1276
Glycoside hydrolases	239	253	249
Carbohydrate esterases	32	32	106
Protease	430	443	480
PHI genes	2844	2892	1953
SMs	41	41	46

Strains PLBJ-1 and PLFJ-1 of P. lilacinum are obtained in this study, strain TERIBC 1 was sequenced in [24] recently.

Among the predicted genes of PLBJ-1, 90.4% were supported by RNASeq data from mycelia cultured in PDB. Both strains exhibited a consistent KOG pattern. Except for the category “General function prediction only”, which was ambiguously sorted to a certain group, the most abundant KOG categories were “Signal transduction mechanisms”, “Posttranslational modification, protein turnover, chaperones”, “Lipid transport and metabolism”, and “Intracellular trafficking, secretion, and vesicular transport” (S2 Fig). A signal peptide analysis showed that 1,410 genes of PLBJ-1 and 1,448 genes of PLFJ-1 encoded putatively secreted proteins.

CAZymes that cleave and build polysaccharides could be required when *P*. *lilacinum* degraded the structural polysaccharide armor of nematode eggshells, such as chitin, during the course of its parasitism. The protease could stop the development of nematode eggs and drastically alter the eggshell structures when applied individually or in combination with chitinases [28, 29]. A detailed examination of the CAZymes and proteases of *P*. *lilacinum* was performed and compared with other fungi, including nematode parasitic fungi (*P*. *chlamydosporia* and *H*. *minnesotensis*), nematode-trapping fungi (*Arthrobotrys oligospora* and *Monacrosporium haptotylum*), entomopathogenic fungi (*T*. *inflatum*, *Beauveria bassiana*, *Cordyceps militaris*, *Metarhizium robertsii*, and *O*. *sinensis*), a mycoparasitic fungus (*T*. *ophioglossoides*), a saprotrophic fungus (*T*. *reesei*) and a plant pathogenic fungus (*F*. *oxysporum*). We identified 53 families containing 239 genes in PLBJ-1 and 55 families containing 253 genes in PLFJ-1 that encoded glycoside hydrolases (GH), which was more than the other fungi (an average of 213) (S4 Table). The most abundant family in PLBJ-1 and PLFJ-1 was GH18, which was represented by 32 and 41 chitinases, respectively, that degrade the chitin present in the chitin protein complex of the nematode eggshell [30]. Consistent with GHs, PLBJ-1 and PLFJ-1 contained relatively more carbohydrate-binding modules (CBMs) (59 and 64, respectively) (S5 Table), which were frequently appended to the enzymes involved in polysaccharide depolymerization. A series of carbohydrate esterase (CE)-encoding genes were also detected in the *P*. *lilacinum* genomes (33 and 32, respectively), including the most abundant sterol esterases (CE10) and cutinases (CE5), which are virulence factors of some plant pathogens [31] (S6 Table). Another major class of CAZymes, the glycosyltransferases (GT), establish natural glycosidic linkages across a broad range of small and macromolecules, and they were represented in the PLBJ-1 genome with 115 members in 32 families and in the PLFJ-1 genome with 124 members in 32 families (S7 Table). These enzymes’ classification demonstrated that they exhibited less variability in ascomycetes than did GHs, a trend that was maintained in a previous analysis [32]. The *P*. *lilacinum* genome contained more proteases (430 and 443, respectively) than other fungi (an average of 396). The largest category of proteases encoded in PLBJ-1 and PLFJ-1 were serine proteases (194 and 198, respectively) (S8 Table), 76 and 81 of which were secreted proteins, respectively. Among the serine proteases, we identified 34 subtilisins (S8) and ten serine carboxyproteases (S10) in the PLBJ-1 genome (36 and 11, respectively, in PLFJ-1), which were reported to be involved in infection and the lethal activity of nematodes [28, 33]. The metalloprotease (108 in PLBJ-1 and 109 in PLFJ-1) and cysteine protease (66 in PLBJ-1 and 68 in PLFJ-1) families also accounted for a significant proportion of the proteases.

A whole genome analysis was conducted against the pathogen-host interaction (PHI) gene database to identify potential virulence-associated genes, under the assumption that the homologue of an experimentally validated pathogenic gene suggested that it played a pathogenic role [34]. We demonstrated that 2,844 (24.1%) and 2,892 (24.6%) proteins of PLBJ-1 and PLFJ-1, respectively, showed sequence similarity to those in the PHI database. Among these proteins, 299 and 317 proteins of PLBJ-1 and PLFJ-1, respectively, were classified as putatively secreted proteins. The KOG functional class distribution of genes related to PHI showed a similar pattern to the whole genome KOG analysis (S2 Fig). The PHI database search yielded 195 CAZymes in PLBJ-1 and 217 in PLFJ-1, 28 and 36 of which were chitinases (GH18), respectively. Of the proteases, 125 in PLBJ-1 and 132 in PLFJ-1 were pathogenic genes according to the PHI database, of which 64 and 72 were identified as secreted proteins, respectively, and these proteins were more likely to function during the infection process [35].

### Phylogenomic relationship and orthologous analysis

**Fig 3:**
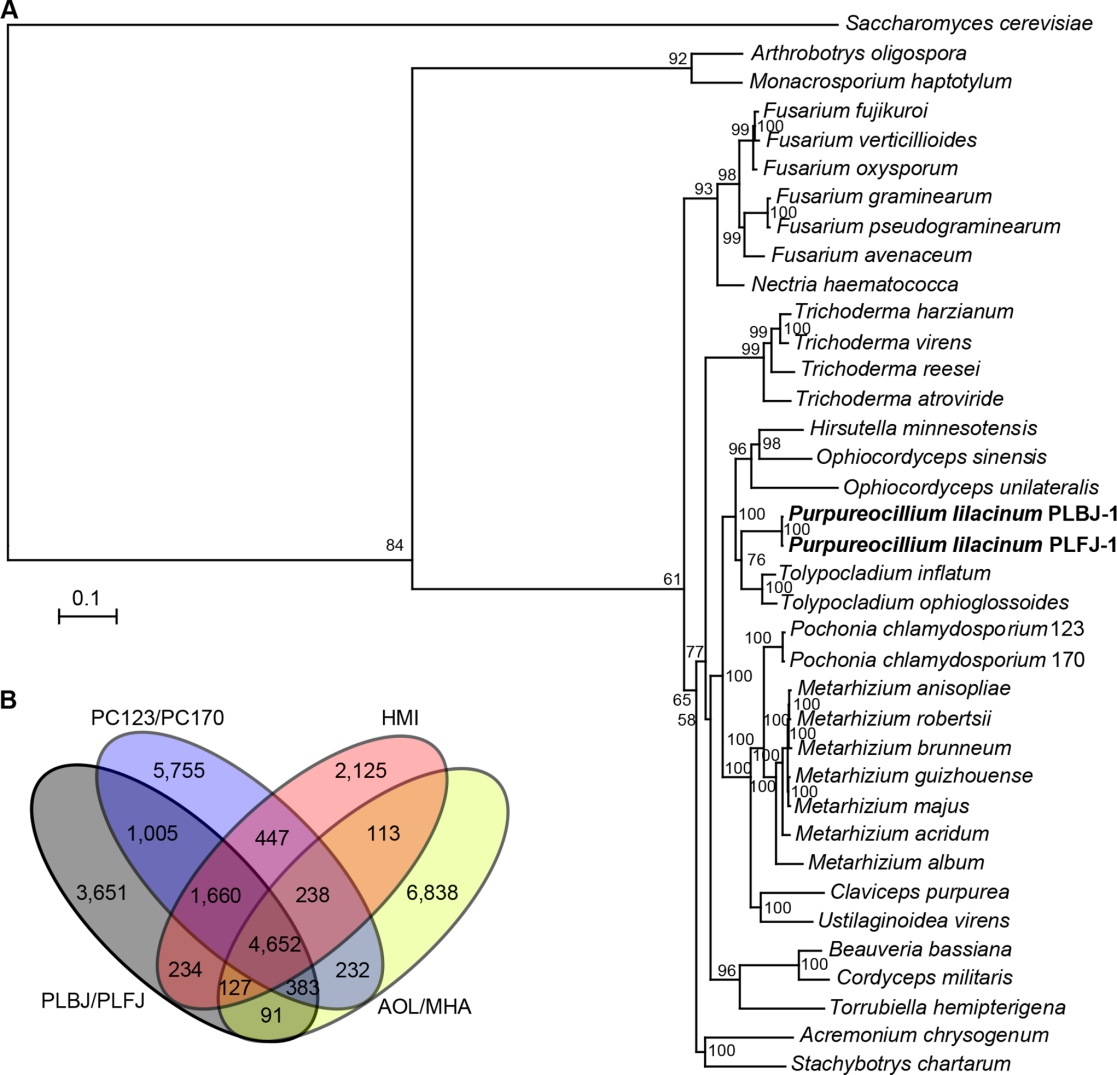
Phylogenomic relationships and orthologous gene clusters. (A) Maximum likelihood phylogeny was computed from a concatenated alignment of 855 groups of single-copy orthologues. Bootstrap values are shown beside the nodes. (B) The number of gene clusters shared by *P*. *lilacinum* with other major associated ecologies. Gray = *P*. *lilacinum* isolates PLBJ-1 and PLFJ-1; blue = nematode egg parasite *P*. *chlamydosporia* isolates 123 and 170; pink = nematode parasite *H*. *minnesotensis*; and yellow = nematode-trapping fungi *A*. *oligospora* and *M*. *haptotylum*.

A phylogenomic tree was constructed based on 855 single-copy orthologues of *P*. *lilacinum* and 34 other filamentous fungi, with *Saccharomyces cerevisiae* as the outgroup. The results verified that *P*. *lilacinum* belongs to Ophiocordycipitaceae, as described by Jennifer Luangsa-ard [8], and it formed a clade with *T*. *inflatum*, *T*. *ophioglossoides*, *O*. *sinensis* [36], *O*. *unilateralis* [37] and *H*. *minnesotensis* (Fig 3A). The inferred phylogeny illustrated that *T*. *inflatum* and *T*. *ophioglossoides* were most closely related to *P*. *lilacinum*, and they diverged after their split with *O*. *sinensis*, *H*. *minnesotensis* and *O*. *unilateralis*. This phylogeny also reinforced the previous analysis that found that the split between Cordycipitaceae (including *B*. *bassiana* and *C*. *militaris*) and Clavicipitaceae (including *P*. *chlamydosporium* and *M*. *anisopliae*) occurred before Ophiocordycipitaceae diverged from Clavicipitaceae (Fig 3A). The three nematode parasitic fungi *P*. *chlamydosporium*, *H*. *minnesotensis* and *P*. *lilacinum* clustered with insect pathogens, indicating that nematode and insect pathogens might share a common ancestor.

A comparative genomic analysis was performed between *P*. *lilacinum* and other nematode-related fungi (the nematode parasites *P*. *chlamydosporia* and *H*. *minnesotensis* and the nematode-trapping fungi *A*. *oligospora* and *M*. *haptotylum*). A total of 17,995 orthologous clusters consisting of 76,151 proteins were identified, of which 4,652 clusters containing 35,972 proteins were mapped to all four of the fungi types (Fig 3B). On the whole, the nematode-trapping fungi, which capture nematodes through an entirely different mechanism compared to *P*. *lilacinum* [26], possessed the largest number of unique gene clusters, although they had a more distant phylogenetic relationship with the other fungi in Hypocreales (Fig 3B). *P*. *lilacinum* contained a large number (3651) of species-specific clusters, while *P*. *lilacinum* shared 7,700, 6,673 and 5,253 clusters with *P*. *chlamydosporia*, *H*. *minnesotensis* and the nematode-trapping fungi, respectively.

### Analysis of paralogous gene families

Lineage-specific expansions could provide material for the evolution of a specific functional system or adaptation in eukaryotes [38]. To study gene family expansions in *P*. *lilacinum*, a comparative genomic analysis of 15 fungal species (PLBJ-1, PLFJ-1, *P*. *chlamydosporia* strain 123, *P*. *chlamydosporia* strain 170, *H*. *minnesotensis*, *A*. *oligospora*, *M*. *haptotylum*, *T*. *inflatum*, *B*. *bassiana*, *C*. *militaris*, *M*. *robertsii*, *O*. *sinensis*, *T*. *ophioglossoides*, *T*. *reesei*, and *F*. *oxysporum*) was performed. In total, 1,963 gene families with more than one gene expansion were identified in both PLBJ-1 and PLFJ-1, of which 1,761 gene families were only present in *P*. *lilacinum*, and some gene families with significant expansion are listed in S9 Table. However, most families were annotated as reverse transcriptases and transposases, and the others were related to transporters or lyases. When the nematode parasitic fungi *P*. *chlamydosporium* and *H*. *minnesotensis* were considered, 2,936 orthologous clusters showed expansion in the five isolates. The largest paralogous expansion contained protein families associated with SMs, such as cytochrome P450s, oxidoreductases, and transporters. In addition, these families also contained transcription factors, glycosyl hydrolases, the hAT family, the majority of which are listed in S10 Table.

### SMs analysis based on the sequence of the *P*. *lilacinum* genome

To evaluate the capability of *P*. *lilacinum* to produce SMs, we searched the genome of PLBJ-1 and PLFJ-1 for biosynthetic genes encoding the four classes of the main SM-associated synthetases, including polyketide synthase (PKS), non-ribosomal peptide synthetase (NRPS), terpene synthase (TS) and dimethylallyl tryptophan synthase (DMATS) [26]. A uniform SM profile with parallel categories and numbers was presented in the two genomes (S11 Table). In total, 13 PKSs, 10 NRPSs, two PKS-like enzymes, 10 NRPS-like enzymes, one DMATS, 4 TSs and one PKS-NRPS hybrid were identified in the PLBJ-1 genome, as described in S11 Table. Compared to sequenced species in Ophiocordycipitaceae, the number of SMs in *P*. *lilacinum* (41) was similar to the 45 SMs in *T*. *ophioglossoides*, 39 SMs in *O*. *unilateralis*, more than 30 SMs in *Ophiocordyceps sinensis*, fewer than 55 SMs in *T*. *inflatum* [39], and 101 SMs in the nematode endoparasitic fungus *H*. *minnesotensis* [26]. These core backbone genes were dispersed among 39 clusters with other enzymes, such as transcriptional regulators, P450s and transporters, as predicted by antiSMASH (antibiotics and Secondary Metabolite Analysis SHell) [40] (S11 Table). According to the BLAST results from the NCBI NR database, no homologues of functionally characterized SMs were detected. Among them, we detected the expression of 29 core genes with FPKM (fragments per kilobase of transcript per million mapped fragments) values > 0.5, using an RNA-seq analysis of PLBJ-1 cultured in PDB medium for 8 days.

A phylogenetic tree was constructed based on the KS domain amino acid sequence of the PKSs in *P*. *lilacinum* and the products of known PKSs, which were divided into three main clades: non-reducing (NR) PKSs, partially reducing (PR) PKSs and highly reducing (HR) PKSs (S3 Fig). VFPBJ_05021, VFPBJ_09342, VFPBJ_09755, and VFPBJ_10843 were predicted as NR PKS-encoding genes, and they shared the highest homology with the non-reducing biosynthetic genes, such as citrinin [41] and griseofulvin [42]. VFPBJ_00212, VFPBJ_02527, VFPBJ_02532, VFPBJ_03442, VFPBJ_05962, VFPBJ _06473, VFPBJ _07567 and VFPBJ_09314 were distributed in the HR PKS clade in close relationship with HR polyketides, such as fumonisin synthase Fum1p [43]. The phylogenetic analysis was consistent with the domain structure analysis of degree of reduction, in which the HR PKS contained the reductive domains KR (keto-reductase), ER (enoyl reductase) and DH (dehydratase), while the NR PKS did not contain these domains (S3 Fig, S11 Table). VFPBJ_05021 and VFPBJ_09342 were grouped with the antibiotics griseofulvin and citrinin with a bootstrap value of 100%, and they shared a common domain structure. This finding suggested that griseofulvin/citrinin or structurally related compounds could be produced by *P*. *lilacinum*. However, we did not detect these compounds when *P*. *lilacinum* was cultured in PDB for 8 days.

Among the 10 NRPSs, six contained one module or an incomplete module, which could encode products with one amino acid. Four NRPSs were multi-module enzymes, which could encode products composed of more than one amino acid. To examine the potential NRPS orthologues of *P*. *lilacinum* and to detect the feasible NRPS evolutionary mechanism in the family Ophiocordycipitaceae, a genealogy was created based on the A-domains from the NRPS of fungi in Ophiocordycipitaceae and several functionally characterized products (S4 Fig). The tree depicted an intricate evolutionary relationship for the NRPS genes. A general trend throughout the tree was that, in Ophiocordycipitaceae, many A-domains clustered with orthologues in other species than with in the same protein. Notably, the 11 A-domains of the cyclosporine synthetases from *T*. *inflatum* clustered separately (S4 Fig, node 3), indicating that other species were incapable of encoding cyclosporine and that its evolution occurred after *T*. *inflatum* diverged from these fungi in Ophiocordycipitaceae.

This phylogenetic analysis of the A-domains for *P*. *lilacinum* detected a series of homologous A-domains: four of the mono-module NRPSs had functionally uncharacterized homologues. VFPBJ_05068 was identified as siderophore synthetase, of which three of the A-domains were grouped with homologues to form a sub-clade (S4 Fig, node 2). The three A-domains of VFPBJ_06596 were grouped with TINF2556, annotated as an ergot alkaloid in *T*. *inflatum*, while TINF2556 contained four modules.

The peptaibiotics, a class of linear NRPSs that are abundant of AIB [44], were clustered into one sub-clade (S4 Fig, node 1), mainly including the peptaibiotics from *T*. *ophioglossoides* [45], *T*. *inflatum* and *P*. *lilacinum*. The ten A-domains from VFPBJ_02539 (identified as the leucinostatin biosynthetic gene *lcsA* in this study), clustered with the ten A-domains from the peptaibiotic TOPH_08469 in *T*. *ophioglossoides*, with bootstrap values of 100%, and a global BLAST analysis revealed that the sequence identity of the two homologues was 65%. Neither orthologue was identified in other species of Ophiocordycipitaceae. The single A-domain of *lcsA* was scattered in the peptaibiotic sub-clade of the tree, while A2, A5 and A6, which activated Leu or related amino acids, were identified in the subsequent study and were grouped together with a bootstrap value of 60%, suggesting that both lineage-specific changes and module duplication contributed to the evolution of the leucinostatin metabolites. In the previous study, A4, A7 and A8 of TOPH_08469 were distributed in a sub-clade enriched in A-domains encoding AIB [45], and our study demonstrated that A4, A7 and A8 of *lcsA* were encoded for AIB.

**Fig 4:**
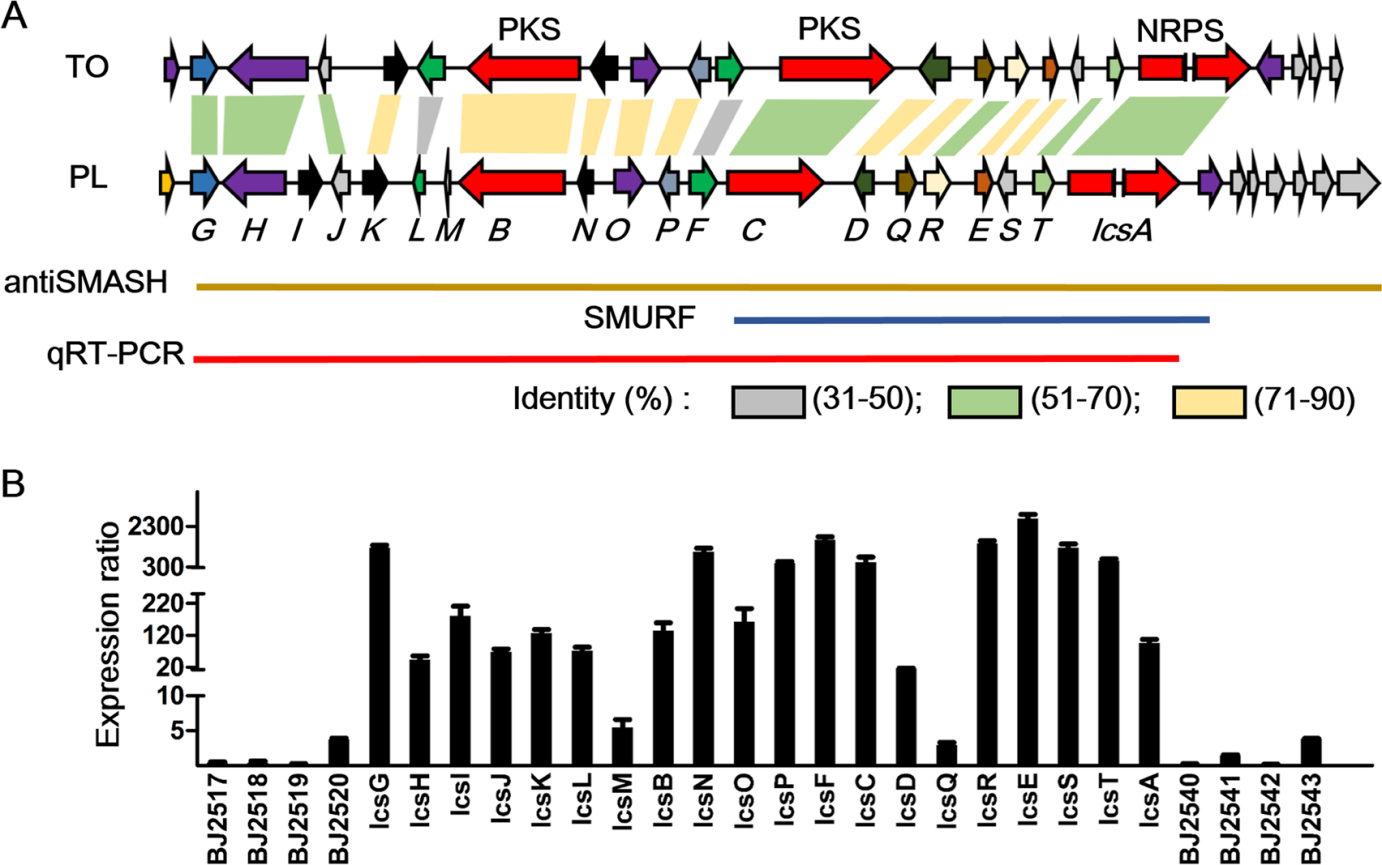
The boundary of the *lcs* cluster in *P*. *lilacinum* with its homologues in *T*. *ophioglossoides*. (A) Horizontal arrows of the same color represent the orthologous genes. The sequence identity between the homologous genes from two fungi is shown by shaded areas with different colors. TO, *T*. *ophioglossoides*; PL, *P*. *lilacinum*. The bars indicate boundaries of the *lcs* cluster predicted by antiSMASH, SMURF, and qRT-PCR. (B) The expression ratio of the genes around *lcsA* when expression in PLBJ-1 cultured in leucinostatin-inducing medium was compared to expression in non-inducing medium. The ratios for different genes demonstrated an extensive range, so the breakpoint was inserted into the Y axis.

In *T*. *ophioglossoides*, the TOPH_08469 gene cluster was predicted to contain 28 genes from TOPH_08452 to TOPH_08478 that were located in an ~124 kb region [45]. A comparative analysis of genes surrounding *lcsA* and TOPH_08469 cluster revealed a high synteny (Fig 4A). VFPBJ_02521 (designed as *lcsG*) shared 68% sequence identity with TOPH_08452, and *lcsA* shared 66% sequence identity with TOPH_08469. Interestingly, no homologues of the genes next to the cluster, VFPBJ_02510 to VFPBJ_02520 and VFPBJ_02540 to VFPBJ_02550, were identified in the *T*. *ophioglossoides* genome. Within the *lcs* cluster, two genes, cytochrome P450 *lcsI* and a protein with unknown function, *lcsM*, did not possess homologues in the TOPH_08469 cluster, while all of the leucinostatin biosynthetic genes in *T*. *ophioglossoides* (TOPH_08452 to TOPH_08469) had homologues within the *lcs cluster*. These results suggested that this nearly 100 kb region might have been horizontally transferred from other fungal or bacterial species. However, leucinostatins have not been reported to be produced by *T*. *ophioglossoides* to date.

### Identification of the NRPS gene *lcsA*, which is involved in the leucinostatin biosynthesis pathway

The lipopeptide leucinostatin A contains ten amide bonds that divide the molecule into 11 moieties, including 4-methylhex-2-enoic acid, 9 amino acid residues and DPD. The property of the mixture of the polyketide and peptide moieties in the leucinostatins indicated a PKS, NRPS or hybrid PKS-NRPS origin. It is logical to consider that a single reducing PKS encodes the 4-methylhex-2-enoic acid, and a NRPS enzyme encodes the remaining portion, as in the models for emericellamide synthesis in *Aspergillus nidulans*[46] and pneumocandin in *Glarea lozoyensis*[47]. Among the multi-module NRPSs in *P*. *lilacinum*, VFPBJ_05068 contains 13 domains grouping into 3 modules, VFPBJ_06596 contains seven domains grouping into three modules, and VFPBJ_11400 contains six domains grouping into two modules. These enzymes were insufficient for the assembly of nine amino acids of leucinostatins. Thus, VFPBJ_02539 was left as the only plausible candidate. VFPBJ_02539 (LcsA) consists of 11,872 amino acids and was encoded by a gene with five introns. The domain structure of LcsA was comprised of 10 C-A-PCP modules and carried the correct number of amino acids for the assembly of leucinostatins. The NRPSpredictor2 [48] offered little insight into the substrates except that the substrates of A1 and A3 were proline and leucine, respectively (S12 Table). Two PKSs, *lcsB* and *lcsC*, located not far upstream of *lcsA*, which could encode 4-methylhex-2-enoic acid, indicated that this cluster is responsible for leucinostatin production. Furthermore, *lcsD* (VFPBJ_02533), located between *lcsA* and the PKSs, was annotated as an acyl-CoA ligase, offering a conceivable route for connecting the fatty acid and peptide.

**Fig 5:**
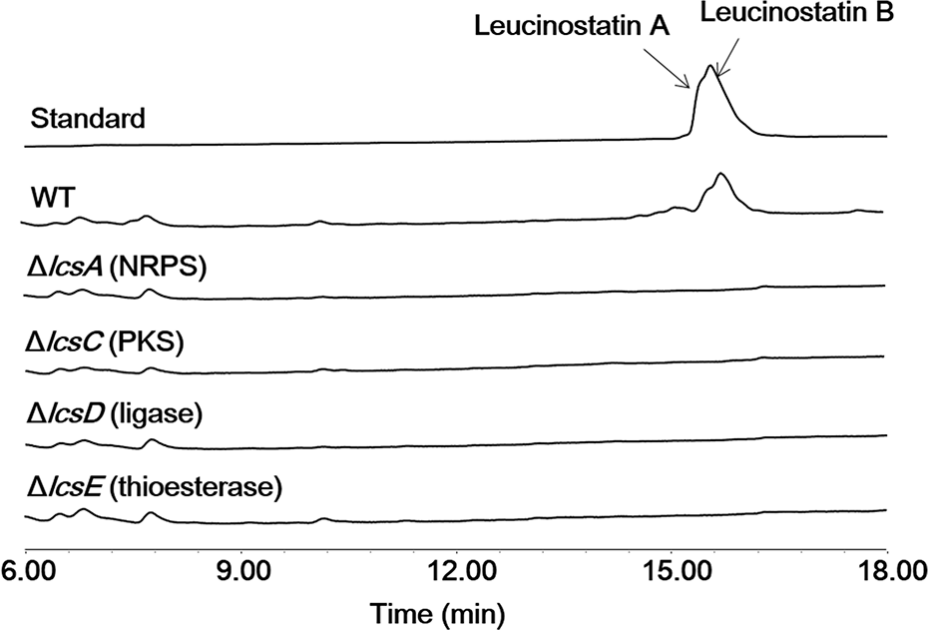
HPLC profiles (UV 210 nm) of culture extracts from the wild type *P*. *lilacinum* strain and mutants when grown in PDB medium. Leucinostatins A and B were detected in the wild type isolate, while they were abolished in Δ*lcsA*, Δ*lcsC*, Δ*lcsD* and Δ*lcsE*.

To verify the associations between the putative *lcsA* and leucinostatins, a gene deletion method was developed for *P*. *lilacinum* based on the previous method for *Fusarium oxysporum*[49], with the G418 sulfate-resistance gene *neo* as the selection marker. A portion of *lcsA* (2,613 bp, including 236 bp upstream of the ORF) was knocked out by double homologous deletion cassettes with the *neo* marker via PEG-mediated transformation, and the resulting G418 sulfate-resistant isolates (S5A and S5B Fig) were verified by diagnostic PCR, using the primers in *neo* and outside the knockout cassette (S5C Fig) (S13 Table). Finally, one mutant (Δ*lcsA*) of PLBJ-1 was isolated with correct PCR amplification products from 320 G418 sulfate-resistance mutants (S5C Fig), and the remaining isolates resulted from ectopic integration of the *neo* gene cassette into the genome. The wild type of *P*. *lilacinum* and the Δ*lcsA* mutant of PLBJ-1 were cultured in PDB medium for 8 days, and the ethyl acetate extracts were analyzed by HPLC-MS. The MS spectrum of the wild type displayed two overlapping peaks at 15.6 and 16.0 min, with *m/z* [M+H]^+^ of 1218.9 and 1204.9, respectively, which were assigned to leucinostatins A and B and were absent in the Δ*lcsA* mutant (Fig 5, S6 Fig). A comparison with the authentic standard confirmed that the missing compounds of the Δ*lcsA* mutant were indeed leucinostatins A and B (Fig 5, S6 Fig). As expected, these results demonstrated the essential roles of *lcsA* in the biosynthesis of the leucinostatins.

### The boundary determination of the leucinostatin biosynthesis gene cluster

Different boundaries of the *lcs* cluster were defined by the SMURF and antiSMASH programs (Fig 4A). Nine genes flanking *lcsA* from *VFPPL_02532* to *VFPPL_02540* spanning 62 Kb were predicted to be in the cluster by SMURF (Secondary Metabolite Unique Regions Finder) [50], while a larger cluster comprising 26 genes from VFPBJ_02521 to VFPBJ_02546, spanning 120 Kb, was predicted by antiSMASH. Therefore, it was necessary to explore the genes that were involved in the pathway using a biological approach.

Changes in the culture medium could impact the general metabolic profile of an organism, based on the “OSMAC” (one strain-many compounds) hypothesis[51]. Indeed, we found that *P*. *lilacinum* produced leucinostatins A and B when cultured with our lab recipe of PDB but did not produce leucinostatins when cultured in PDB-BD (see the Materials and Methods section). This result provided clues to identify the boundary of the *lcs* cluster using producing versus non-producing media. qRT-PCR analysis was conducted to compare the expression patterns of genes flanking *lcsA* when PLBJ-1 was grown in the two types of media for 8 days. Furthermore, RNA-Seq of PLBJ-1 under leucinostatin-inducing conditions (PDB medium) was performed.

As expected, the expression level of NRPS *lcsA* when *P*. *lilacinum* was grown in leucinostatin-inducing medium was upregulated 95-fold, compared to those grown in non-inducing medium (Fig 4B). The genes downstream of *lcsA*, including the putative transporter ABC gene VFPBJ_02540, did not display a higher expression level in the leucinostatin-inducing medium, indicating that they were not involved in the leucinostatin biosynthesis pathway. Correspondingly, the RNA-Seq expression profile during leucinostatin production showed a low FPKM value of VFPBJ_02540 (2.01) (S7A Fig), while the FPKM value of *lcsA* was 65.4. These results indicated that the 3’ edge of the cluster was *lcsA*. The genes upstream of *lcsA* from VFPBJ_02520 to VFPBJ_02538 (*lcsT*) were upregulated at different levels in the leucinostatin-inducing medium. A 16- to 2692-fold increase in expression was observed (Fig 4B), except for three genes, VFPBJ_02520, *LcsM*, and *lcsQ*, which showed less than 10-fold increase and low FPKM values in the transcriptional data (S7A Fig). VFPBJ_02520 was annotated as a phosphohydrolase that appeared to be involved in nucleic acid metabolism and signal transduction, instead of secondary metabolism[52]. Thus, we speculated that the 5’ boundary of the cluster was VFPBJ_02521 (*lcsG*). To support this hypothesis, the expression patterns of the genes flanking the cluster were analyzed using qRT-PCR analysis in wild type PLBJ-1 and Δ*lcsA* grown in leucinostatin-inducing medium. We observed an increase in the expression of wild type *P*. *lilacinum* ranging from four- to 79-fold (S7B Fig). Thus, a series of genes from VFPBJ_02521 to VFPBJ_02539, designated as *lcsA* to *lcsT*, included the core enzymes, modifying enzymes and transporter enzymes coding for the biosynthesis of leucinostatins (Fig 4A, Table 2).

**Table 2:** Description of the genes in the leucinostatin biosynthetic cluster.

Gene ID	Name	Length	Conserved domain	Deduced function
VFPBJ_02519		270	PhyH	Epoxidase subunit
VFPBJ_02520		214	HD	Phosphohydrolase
VFPBJ_02521	*lcsG*	468	Methyltransf_2	O-methyltransferase
VFPBJ_02522	*lcsH*	1552	ABC_tran	ABC transporter
VFPBJ_02523	*lcsI*	516	p450	Cytochrome P450
VFPBJ_02524	*lcsJ*	309	4HBT_2	Thioesterase-like
VFPBJ_02525	*lcsK*	548	p450	Cytochrome P450
VFPBJ_11786	*lcsL*	228	bZIP	Transcriptional regulator
VFPBJ_02526	*lcsM*	82		Hypothetical protein
VFPBJ_02527	*lcsB*	2507	KS-AT-DH-MET-ER-KR	PKS
VFPBJ_02528	*lcsN*	336	p450	Cytochrome P450
VFPBJ_02529	*lcsO*	593	ABC1	ABC transporter
VFPBJ_02530	*lcsP*	387	Aminotran_4	Aminotransferase
VFPBJ_02531	*lcsF*	552		Transcriptional regulator
VFPBJ_02532	*lcsC*	2332	KS-AT-DH-MET-ER-KR-ACP	PKS
VFPBJ_02533	*lcsD*	386	AMP-binding domain	Acyl-CoA ligase
VFPBJ_11787	*lcsQ*	389	tRNA-synt_2c	tRNA synthetases
VFPBJ_02534	*lcsR*	400	Lactamase_B	Zn-dependent hydrolases
VFPBJ_02535	*lcsE*	316	Thioesterase	Thioesterase
VFPBJ_02536	*lcsS*	243		Hypothetical protein
VFPBJ_02538	*lcsT*	269	CC3_like_SDR_a	Epimerase
VFPBJ_02539	*lcsA*	11872	PCP-(C-A-PCP)*10-NAD	NRPS
VFPBJ_02540		1444	ABC2_membrane	ABC transporter
VFPBJ_02541		594	Sec14p-like	Phosphatidylinositol transfer protein

VFPBJ_02519, VFPBJ_02520, VFPBJ_02540 and VFPBJ_02541 are genes flanking *lcs* cluster.

### Identification of the leucinostatin biosynthetic pathway

Considering the structural similarities of leucinostatin A with emericellamide A [53] and pneumocandin [47], we reasoned that a similar biosynthetic mechanism might be required to form the skeletons of lipopeptides and peptides. As reported, a single module polyketide synthase iteratively catalyzes the formation of the linear polyketide chain; in daptomycin [54] and echinocandin B [55], acyl-CoA ligase converts the fatty acid to fatty acyl CoASH; in compound W493 B [56], a thioesterase was proposed to hydrolyze the thiol bond and shuttle the product to the first module of NRPS. To determine whether the same enzymes play critical roles in the leucinostatin biosynthesis pathway, we disrupted the PKS (*lcsC*), ligase (*lcsD*) and thioesterase (*lcsE*)-encoding genes in the cluster by homologous recombination (S5A Fig) and verified the mutants by PCR amplification (S8A Fig). After culturing the fungi in PDB medium and comparing the extracts with the PLBJ-1 wild type and Δ*lcsA* by HPLC-MS, we showed that leucinostatins A and B disappeared in Δ*lcsC*, Δ*lcsD* and Δ*lcsE*, similar to Δ*lcsA* (Fig 5, S6 Fig).

### Overexpression of the transcription factor *lcsF*

A powerful approach to enhancing the production of leucinostatins was to express transcription factors constitutively that were used for other SMs [57]. *lcsF* encodes a putative transcription factor with a bZIP domain structure, and it is associated with secondary metabolism [58]. To assess the function of *lcsF*, we cloned it into the KSTNP vector under the control of the TrpC promoter. The resulting plasmid, KSTNP-OE*lcsF*, was randomly integrated into the genome of wild type *P*. *lilacinum* (S8B Fig). The positive transformants were screened by G418 sulfate and were diagnosed by PCR amplification of the expression cassette (S8C Fig). Transformants with an intact overexpression cassette were cultured in leucinostatin-inducing PDB medium for 8 days. The expression level of *lcsF* in the mycelia was analyzed by qRT-PCR, and six of ten transformants demonstrated more than 20-fold upregulation. Finally, three transformants without changes in their physiological indices were selected for the downstream test. As expected, all 20 genes in the cluster were upregulated to some extent by *lcsF*, with the exception of *lcsL* and *lcsP*, which were downregulated three- and five-fold (S9A Fig). In addition to the 30-fold increase in *lcsF* expression, the expression of the three PKS/NRPS synthase encoding-genes (*lcsB*, *lcsC* and *lcsA*) were increased by ~3- to 4-fold. For O-methyltransferase (*lcsG*), ABC transporter (*lcsH* and *lcsO*), thioesterase (*lcsE*), epimerase (*lcsT*) and the unknown function genes *lcsM* and *lcsS*, we observed ten-fold or higher upregulation. The other genes in the cluster displayed a two- to ten-fold increase in expression. Genes adjacent to the *lcs* cluster, VFPBJ_02520 and VFPPL_02540, were downregulated three-fold. After the wild type and OE::*lcsF P*. *lilacinum* were grown in PDB medium with shaking for 8 days, the resulting HPLC profile showed that the titers of leucinostatins A and B were elevated by at least 50% (S9B Fig). These results provided evidence that the pathway-specific transcription factor *lcsF* was capable of regulating the entire gene cluster and leucinostatin biosynthesis, further verifying the boundary of this cluster.

### Antagonism against the oomycetes of *P*. *lilacinum* depending on leucinostatins

**Fig 6:**
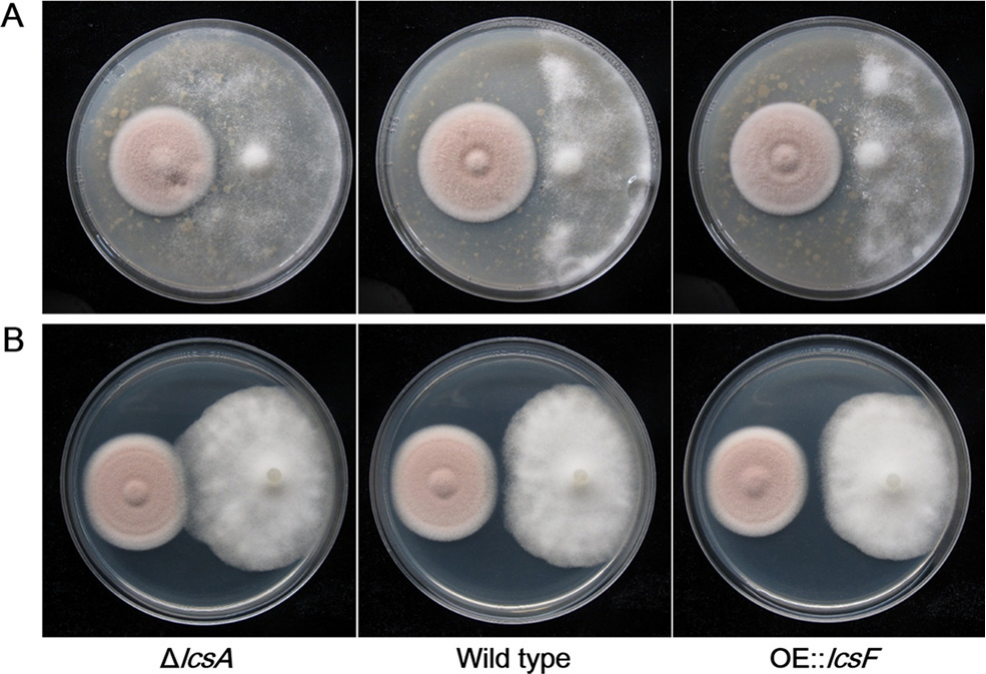
The role of leucinostatins in antagonism between *P*. *lilacinum* and *Phytophthora*. (A) Cocultivation of *P*. *infestans* and wild type, Δ*lcsA* and OE::*lcsF P*. *lilacinum* on rye agar medium. (B) Cocultivation of *P*. *capsici* and wild type, Δ*lcsA* and OE::*lcsF P*. *lilacinum* on PDA medium.

The deletion and overexpression of the genes in the *lcs cluster* had no apparent effects on the fungal hyphae or spore phenotypes of *P*. *lilacinum* and did not cause any growth defects. It is well known that leucinostatins are antibiotics used to combat fungi and bacteria. Here, we found that leucinostatins contributed to the inhibition of oomycetes, which had not previously been reported. The growth of *P*. *infestans* and *P*. *capsici* was inhibited in a confronting incubation with wild type *P*. *lilacinum* and OE::*lcsF*, while the inhibition disappeared when they were grown in a confronting incubation with Δ*lcsA* (Fig 6). Similar to Δ*lcsA*, *P*. *infestans* could grow normally in a confronting incubation with Δ*lcsC*, Δ*lcsD* and Δ*lcsE* (S10 Fig). The results indicated that leucinostatins A and B inhibited the growth of some oomycetes. A gradient inhibitory zone was explored with leucinostatins A and B in different concentrations to find quantitative evidence of inhibition against *P*. *infestans* (S11 and S12 Figs).

## Discussion

*P*. *lilacinum* is one of the most important endo-parasites of plant nematodes. We obtained the genome sequence of two *P*. *lilacinum* strains and compared them with other nematode parasites, nematode-trapping fungi, insect parasites, a mycoparasitic fungus, a saprotrophic fungus and a plant pathogen. This method provided insights into the life strategy and evolution of nematode endoparasites.

Major gene families (GH, protease, SMs) could corroborate each other for the three *P*. *lilacinum* strains (PLBJ-1, PLFJ-1 and the published TERIBC 1). However, TERIBC 1 was predicted to encode more CEs and fewer PHI genes in contrast with PLBJ-1 and PLFJ-1 (Table 1). The *lcs* cluster was also detected in the TERIBC 1 genome. The genomic sequence identity of the *lcs* cluster (*lcsG* to *lcsA*) between PLBJ-1 and TERIBC 1 was 98.0%, and the sequence identity was 99.0% between PLBJ-1 and PLFJ-1, with the syntenic relationships shown in S13 Fig.

Although fungi have been screened for activity as bio-control agents against *P*. *infestans*, the biological control of late blight is dominated by bacterial antagonists. Microbial compounds known as biosurfactants [59] are believed to participate in the process. For example, the cyclic lipopeptide massetolide A produced by *Pseudomonas fluorescens* exhibited destructive effects on the zoospores of oomycetes [60]. The inhibition of *P*. *infestans* by leucinostatins provided the basis for their chemical application in agriculture and for further biological studies of the antagonist *P*. *lilacinum* on oomycetes, which had not been researched previously. Leucinostatins also demonstrated inhibition against *P*. *capsici* [61], another oomyceteous plant pathogen, while the antagonism of *P*. *lilacinum* against *P*. *capsici* seemed inferior to that against *P*. *infestans*, as shown in Fig 6.

The phylogenomic analysis revealed that *P*. *lilacinum* was a member of Ophiocordycipitaceae, Hypocreales, which includes fungi engaged in various lifestyles, and it was not related to the previously considered *Paecilomyces* in Sordariales. This species’ closest relatives, *T*. *inflatum* and *T*. *ophioglossoides*, are insect and fungal parasites (Fig 3A), supporting the viewpoint that parasitism might occur due to the formation of novel genes that could be acquired through horizontal transfer or gene duplication and could play specific roles during host infection [62]. Moreover, these results indicated that the nematode pathogens had a strong link with insect pathogens and were distantly related to nematode-trapping fungi, as previously described in [25] and [26]. The large number of hydrolytic enzymes, particularly GHs and proteases, putatively secreted proteins and pathogenesis-related proteins in *P*. *lilacinum* support its various lifestyles as it encounters diverse nutrient resources [63]. Chitin and proteins comprise a significant proportion of the nematode and insect surface, the degradation of which requires serine proteases and chitinases.

The development of natural compounds from bio-control fungi have recently attracted considerable interest because the production of nematode-toxic SMs could also be a strategy for fungi to infect nematodes [64]. Next-generation sequencing technologies are becoming an essential tool for identifying novel genes for metabolite biosynthesis in fungi. The genome sequence of *P*. *lilacinum* revealed the potential to produce a rich repertoire of SMs, including 41 core enzymes. Most of the PKS enzymes could be clustered with the PKS enzymes encoding bioactive polyketides when the PKS tree was constructed based on the KS domains (S3 Fig). The NRPS enzyme tree based on the A-domains demonstrated that some of NRPSs of *P*. *lilacinum* possessed homologues in closely related species (S4 Fig). However, none of the enzymes’ biosynthetic function have been validated previously. The polyketide compounds acremoxanthone C and acremonidin A belong to the xanthone−anthraquinone heterodimer that was recently isolated from *P*. *lilacinum*. Structurally related heterodimeric compounds, such as acremoxanthones A and B [65] and xanthoquinodin B3 [66], have been isolated from the genera *Acremonium* and *Humicoma*. This category of compounds and their derivatives have remarkable biological and medicinal activities, and their total synthesis has garnered attention worldwide. Unfortunately, there still exist limitations in the current synthesis methods [67], and metabolite regulation based on molecular biosynthesis is not available because the biosynthetic genes have not been identified. One or more non-reducing PKSs (S3 Fig) might be involved in the biosynthesis of acremoxanthone C and acremonidin A, according to their structures.

**Fig 7:**
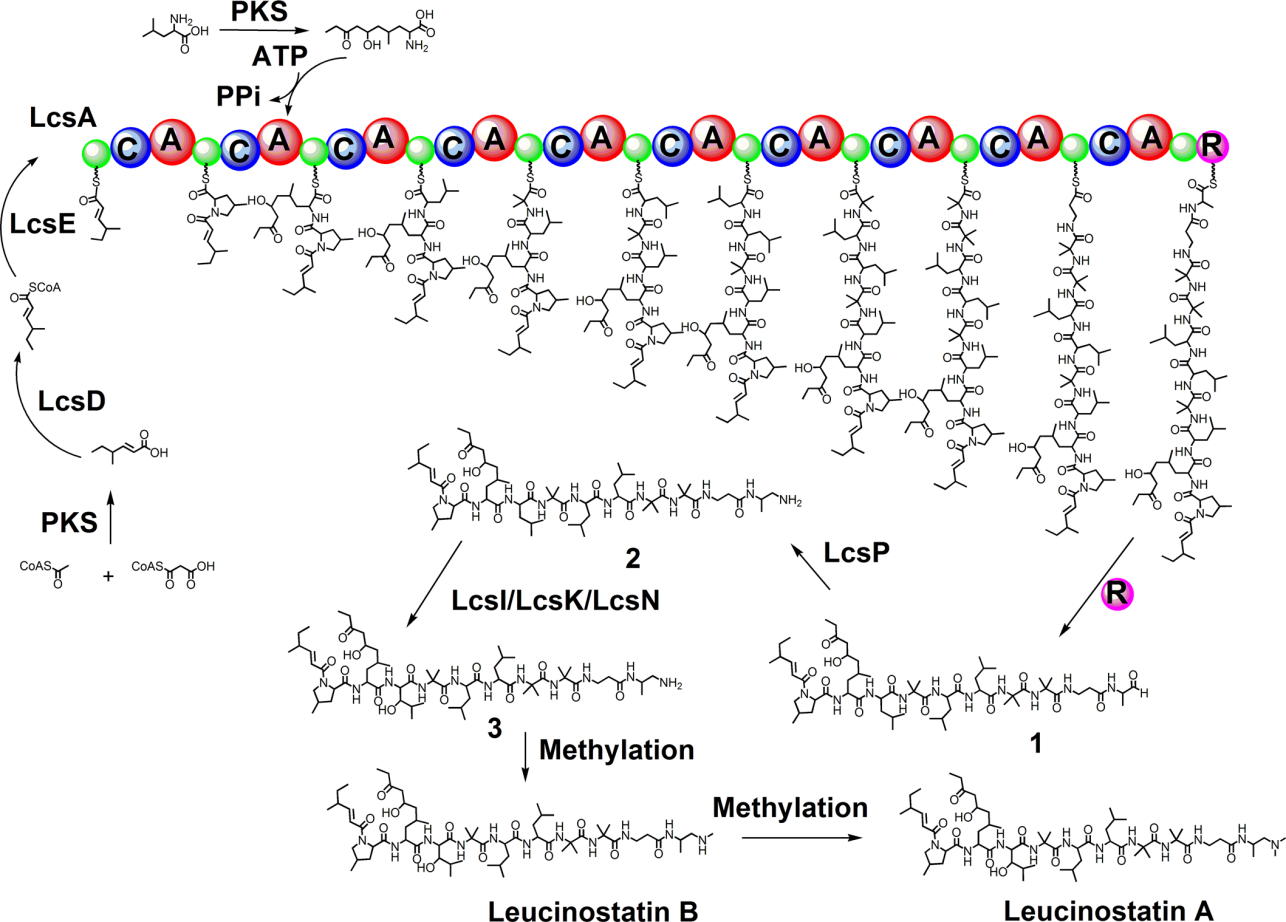
A putative biosynthetic pathway for leucinostatin A.

By analyzing the genes located in the leucinostatin biosynthetic cluster, combined with the HPLC-MS analysis of gene deletion mutants (Fig 5), we were able to propose a putative biosynthetic pathway for leucinostatins (Fig 7). This hypothetical biosynthesis initiated with the assembly of 4-methylhex-2-enoic acid by a reducing PKS. However, two reducing PKS encoding genes with 38% sequence identity are present in the cluster, and both contained KS, AT, DH, cMT, ER, KR and ACP domains. We excluded the possibility of a partnership between the two PKSs in a sequential manner or a convergent manner, as has been reported for asperfuranone [68] and azaphilone A [69], based on their structures. Moreover, in the biosynthesis of chaetoviridins and chaetomugilins from *Chaetomium globosum*, a PKS cazF (KS-AT-DH-cMT-ER-KR-ACP) encoded an intermediate 4-methylhex-2-enoic acid [70]. The protein sequence identities between cazF and lcsB/lcsC were both 29%; thus, we could not estimate which PKS was responsible for 4-methylhex-2-enoic acid. The results of RNA-seq and qRT-PCR indicated that both genes contributed to leucinostatin synthesis. The deletion of *lcsC* interrupted leucinostatin biosynthesis, which confirmed that *lcsC* is essential for the synthesis of leucinostatins. Due to the difficult genetic manipulation, we failed to obtain *lcsB* deletion mutant.

The lipopeptide pathways and organizations of their clusters have some striking commonalities. We got clues from the lipopeptides echinocandin B [55], pneumocandin [47] and emericellamide [53]. The polyketide residue might be transferred to the NRPS LcsA, mediated by two additional putative enzymes, acyl-CoA ligase (LcsD) and thioesterase (LcsE). The linear polyketide carboxylic acid, which was released from PKS, was converted to a CoA thioester by LcsD, and then LcsE hydrolyzed the thiol bond and shuttled the polyketide intermediate to LcsA. 4-Methylhex-2-enoic acid was not detected in the culture of the Δ*lcsD* isolate, indicating that the triketide might be sticked in the PKS enzyme to prevent its release until the ligase is added for the reaction.

The phylogenetic analysis of the ten A-domains of LcsA revealed that LcsA_A2, LcsA_A5, LcsA_A6 and LcsA_A3 were grouped into one clade, and LcsA_A4, LcsA_A7 and LcsA_A8 were grouped into another clade (S14 Fig). The conserved domains are believed to have evolved through module duplication, and they activate similar amino acid structures [71]. In the plausible model for leucinostatin synthesis, A5 and A6 incorporated leucine, A2 incorporated AHyMeOA, the structure of which is equal to a hydroxyl-3-pentone extending at the leucine, and A3 incorporated 3-hydroxyl leucine. A4, A7 and A8 incorporated AIB. Thus, the structural similarity of amino acids activated by conserved A-domains verified that the 4-methylhex-2-enoic acid moiety in the leucinostatins was assembled by a discrete enzyme, instead of LcsA. The C domain of the first module catalyzed the condensation of 4-methylhex-2-enoic acid and MePro carried by domain A1, followed by successive condensations of nine amino acids to trigger the elongation of the linear peptide. Next, the peptide scaffold would be released by the NAD(P)H-dependent R domain (thioester reductase) at the C-terminal region of LcsA.

In the leucinostatin biosynthetic pathway, it is intriguing that the DPD residue at the C-terminus of leucinostatin A was neither an amino acid nor a carboxy acid, which are incapable of being activated by the A-domain and converting to amino acyl adenylate [72]. The DPD seems to be a modified form of amino acids, whereas the primary form of this moiety cannot be determined based on the domain sequence. However, we could deduce a possible pathway for the modification of the last amino acid according to the structure and the function of the genes in the cluster (Fig 7). Originally, an Ala was likely incorporated into the decapeptide skeleton by the A10 domain, which was attached to LcsA via a thioester bond; subsequently, the R domain released this intermediate product. The NAD(P)H-dependent R domains reductively catalyzed to produce linear aldehyde **1** by off-loading peptide thioesters, following completion of the peptide skeleton, as presented in previous studies [73, 74]. Linear aldehydes frequently occurred as intermediates and underwent subsequent reactions, such as macrocyclization, to yield the imine product koranimine [75]; further reduction yielded myxochelin A or transamination to form an amine myxochelin B by aminotransferase [76]. Regarding the leucinostatins, we speculated that aldehyde **1** would go through a transamination reaction to form compound **2**, which was accomplished by the putative aminotransferase *lcsP*.

In this pathway, the unhydroxylated leucine of intermediate **2** undergoes hydroxylation to form compound **3**. Three putative cytochrome P450-encoding genes (*lcsI*, *lcsK* and *lcsN*) within the cluster alternatively might catalyze this modification. Another scenario equivalent to this pathway for leucinostatin A synthesis was that a leucine was hydroxylated prior to its incorporation into the peptide. In all likelihood, the varying extents of methylation of compound **3** catalyzed to form leucinostatins A and B. It is worth mentioning that, had the methylation reaction not occurred, compound **3** might be the ultimate precursor of leucinostatin C, which is compound in leucinostatin family isolated from *P*. *marquandii*.

The AHyMeOA in leucinostatin A activated by the A2 of *lcsA* was regarded as a ramification of leucine because leucine is located at this position in leucinostatins C, T, F, D and H, although these compounds were not detected in the PLBJ-1 culture. Based on its structure and the presence of redundant PKSs within the cluster, alternative PKS could be involved in synthesizing the carbon chain. In addition, the leucinostatins contained the nonproteinogenic MePro, incorporated in the synthesis of nostopeptolides in *Cyanobacteria* [77]. A zinc-dependent dehydrogenase, nosE, and a P5C reductase, nosF, were involved in the oxidation and subsequent cyclization of leucine to form MePro, and the presence of *nosE* and *nosF* recently led to screening for novel MePro-containing peptides [78]. In the pneumocandins from *Zalerion arboricola*, feeding experiments established that leucine was cyclized to produce 3-hydroxy-4-methylproline, whereas MePro might be an intermediate [79]. It was reasonable to assume that the MePro in the leucinostatins originated from leucine cyclization. Although homologues of *nosE* nor *nosF* were not present in the *lcs cluster*, it was plausible that MePro biosynthesis, engaged in a separate pathway, was independent of leucinostatin synthesis. Another nonproteinogenic amino acid, β-Ala, was present in leucinostatins and activated by the A9 of *lcsA*. A previous study of the destruxins in *Metarhizium* proposed that the aspartic acid decarboxylase dtxS4 triggered the decarboxylation of aspartic acid into β-Ala, as a substrate for the assembly line [80]. A genome-wide blast search for genes encoding aspartic acid decarboxylases in PLBJ-1 revealed the presence of two candidate genes, VFPBJ_01400 and VFPBJ_10476, with 68% and 61% sequence identity to dtxS4, respectively, which could have catalyzed the biosynthesis of β-Ala in leucinostatins.

### Conclusions

The genomes of *P*. *lilacinum* strains PLBJ-1 and PLFJ-1 were sequenced, completely assembled, annotated, and comparatively analyzed with related fungi. Phylogenomic analysis showed that *P*. *lilacinum* was most closely related to *T*. *inflatum* and *T*. *ophioglossoides*, and the cluster of nematode parasitic fungi and insect pathogens indicated their common origin. PKS and NRPS-encoding genes were thoroughly characterized and analyzed by phylogenetic analysis, from which we found that *lcsA* was specific to *P*. *lilacinum* and *T*. *ophioglossoides*. Furthermore, *lcsA* was proved to be responsible for leucinostatin biosynthesis by homologous deletion. The boundary of the *lcs* cluster was identified by comparison of gene expression levels when *P*. *lilacinum* was cultured in leucinostatin-inducing and non-inducing medium as well as RNA-Seq analysis. Disruption of *lcsC*, *lcsD* and *lcsE* demonstrated the critical roles of PKS, acyl-AMP ligase and thioesterase in the biosynthetic pathway of leucinostatins. Overexpression of the transcription factor *lcsF* increased the production of leucinostatins A and B through regulated expression levels of genes in the *lcs* cluster. We also demonstrated that leucinostatins could enable the fungus with antagonistic activity against the oomycetes.

## Materials and Methods

### Fungal strains, vectors and reagents

The leucinostatin-producing *P*. *lilacinum* strain PLBJ-1 (CGMCC3.17492) was isolated from tomato roots in Beijing, China, and PLFJ-1 (CGMCC3.17493) was isolated from tomato roots in Fujian, China. Both strains were sequenced to obtain the common features of *P*. *lilacinum* and to ensure the information accuracy of the *lcs* cluster. PLBJ-1 was used as the wild type recipient for the subsequently genetic manipulations because PLFJ-1 was insensitive to the antibiotics used as selection markers. *P*. *infestans* and *P*. *capsici* were maintained at the Chinese Academy of Agricultural Sciences. The pKOV21 vector used for homologous deletion and the KSTNP vector used for overexpression came from Prof. Youliang Peng, China Agricultural University. The leucinostatin A standard came from Bioaustralis, Inc. (NSW, AUS). G418 sulfate was purchased from Amresco, Inc. (OH, USA).

### Medium and culture conditions

The non-inducing PDB-BD medium (Potato Dextrose Broth) came from Becton, Dickinson and Company (NJ, USA). Leucinostatin-inducing PDB medium was prepared in the lab. Briefly, 200 g of potatoes were boiled for 30 min, and then 20 g of glucose were dissolved into the filtrate and diluted to 1 L. The rye agar medium contained 50 g of crushed rye, 20 g of sucrose and 15 g of agar per liter. PDB cultures with 1×10^5^ conidia per mL of PLBJ-1 were grown at 28°C on a shaker at 150 rpm for 8 days before DNA/RNA isolation.

### DNA and RNA isolation

The mycelium tissues of the PLBJ-1 and PLFJ-1 isolates were harvested via filtration. Genomic DNA was isolated using a Qiagen DNeasy kit, according to the manufacturer’s protocol. The PLBJ-1 tissue for RNA isolation was grown in the leucinostatin-inducing medium. RNA was extracted using TRIZOL reagent (Invitrogen, USA) following the manufacturer’s protocol.

### Genome assembly, gene prediction and RNA-seq analysis

The raw sequencing data (Illumina HiSeq 2000) from the PLBJ-1 and PLFJ-1 strains were generated by BGI-Shenzhen (China) and Berry Genomics Co., Ltd. (China), respectively. A total of 13.27 Gb bases for the PLBJ-1 strain from three libraries, with average insert sizes of 165 bp, 758 bp and 5,490 bp, were obtained, and 5.88 Gb bases for the PLFJ-1 strain from two libraries with the average insert sizes of 175 bp and 4,760 bp were obtained. Both of the genomes were assembled using ALLPATHS-LG revision 42305 [81]. The repeat sequences were identified as previously described [82], based on *de novo* and homology methods. For the *de novo* method, Piler [83] and RepeatScout, version 1.0.5 [84], were used to construct the repeat sequence families; then, RepeatMasker, version 4.0.5, was used for repeat analysis. For the homology method, the sequence families from Repbase, version 19.06 [85], were used for annotation by performing RepeatMasker analysis.

For gene prediction, the Augustus algorithm, version 2.7 [86], identified 11,404 and 11,554 complete genes for the PLBJ-1 and PLFJ-1 strains, respectively, and the GeneMark-ES algorithm, version 2.3f [87], discovered 11,001 and 11,070 complete genes for the PLBJ-1 and PLFJ-1 strains, respectively. The comparison showed that 9,509 and 9,562 genes of the PLBJ-1 and PLFJ-1 strains were predicted by both Augustus and GeneMark-ES. These consensus genes were considered to be high quality predicted genes and were used in this study. The additional 2,264 and 2,201 genes of the PLBJ-1 and PLFJ-1 strains were obtained according to the method in [82]. EuGene, version 4.1 [88], was used to integrate multiple sources, including transcription start sites identified by Netstart [89], homologous proteins identified from the Swiss-Prot database, version 2015-07-22, by BLAST, version 2.2.26, the assembled transcripts generated by IDBA-tran, version 1.1.1 [90], and the exon junctions identified from RNA-seq by Tophat, version 2.0.13 [91]. The gene expression values were presented by the expected FPKMs using Cufflinks, version 2.2.1 [92], based on the Tophat [91] analysis.

### Protein family classification

Proteins were annotated by aligning their sequences to the NCBI fungi refseq, version 2015-07-10, and SwissProt, version 2015-07-22, with an E-value cutoff of 1e-5 using BLASTP. In addition, the Pfam database, version 27.0, was used for domain annotation by HMMER, version 3.1b1 (http://hmmer.janelia.org/). The putative proteins were further classified by Gene Ontology (GO) [93], using Blast2Go [94], and the euKaryotic Clusters of Orthologous Groups (KOG) [95], using BLAST (E-value of 1e-5). The Web server of the CAZymes Analysis Toolkit (CAT) [96] was used to identify CAZymes in *P*. *lilacinum* with E-values ≤ 1e-50.

The proteases were discovered by the MEROPS batch BLAST online server [97]. Proteins with sequences that matched the cytochrome P450 genes [98] with E-values ≤ 1e-50 were annotated as P450 enzymes. Candidate pathogenic factors were predicted by sequence alignment against the Pathogen Host Interactions (PHI) database, version 3.5 [99], with E-values ≤ 1e-50. In addition, the secretomes were identified based on recognizing the signal peptide and transmembrane sequences. Proteins were considered to be secreted proteins if the signal peptides were identified by at least two methods among SignalP, version 4.0 [100], TargetP, version 1.1 [101], Phobious, version 101 [102], and Predisi [103], and transmembrane sequences were not identified by at least one of the methods among SignalP, Phobious and TMHMM, version 2.0c [104].

### Orthologous and phylogenomic analysis

Orthologous groups of genes from *P*. *lilacinum* and the other fungi listed in S14 Table were detected by OrthoMCL, version 2.0.9 [105], and then were filtered to identify the single copy orthologues. The single copy orthologues were aligned with MUSCLE [106]. The poor alignment regions of the concatenated sequences were removed using Gblock, version 0.91b [107], and then the high quality sequences were used for the maximum likelihood phylogeny analysis with the Dayhoff model implemented in the TREE-PUZZLE program [108]. Bootstrap support value was calculated by analyzing 1,000 replicates.

### Phylogenetic analysis of the PKS and NRPS genes

The secondary metabolite genes were discovered by performing SMURF [50] and antiSMASH [40] analyses. PKS and NRPS domain structures were characterized by antiSMASH and Pfam, or were visually identified by multiple alignments. The KS domains extracted from PKS and the A-domain from NRPS were aligned by MUSCLE [106], and then a maximum likelihood phylogeny was constructed by treeBeST (http://treesoft.sourceforge.net/treebest.shtml) using 1,000 bootstrap replicates.

### qRT-PCR analysis

Three biological replicates were performed for each analysis of the relative expression levels. The cDNAs were synthesized with a TIANScript Ⅱ RT Kit (TIANGEN, China). The cDNA was analyzed by qRT-PCR using SYBR Premix Ex Taq (TAKARA, Japan) on a BIO-RAD CFX96 (BIO-RAD). The housekeeping actin gene designed from VFPBJ_07912, which was similar to the reported GU299860.1, was used for normalization. The relative expression values were calculated using the 2^-∆∆Ct^ method. The primers are listed in S13 Table.

### Molecular genetic procedures

Polyethylene glycol-mediated protoplast transformation of PLBJ-1 was performed as previously reported [49, 109], with the following modifications: the protoplast was produced by 20 gL^−l^ Driselase (Sigma) digestion for 4 h at 31°C. The regeneration medium was PDA medium containing G418 sulfate (400 μg/L), supplemented with molasses (10 g/L), saccharose (0.6 M), yeast extract (0.3 g/L), tryptone (0.3 g/L), and casein peptone (0.3 g/L) [10]. The construction of knockout and overexpression plasmids originated from pKOV21 and KSTNP, and the primers are listed in S13 Table. A quick method for isolating the fungal genomic DNA was developed to screen for a large number of transformants. Briefly, a nip of mycelia was transferred to 50 μL of NaOH (50 mM) and was incubated at 95°C for 20 min. The solution was directly used for PCR amplification after 5 μL of Tris-HCl (1 M) were added to neutralize the base.

### Culture extraction and HPLC-MS profiling

Cultures of 1×10^5^ conidia per mL of *P*. *lilacinum* and its mutants were grown in leucinostatin-inducing PDB medium at 28°C on a shaker at 150 rpm for 8 days. Culture medium (7.5 L) was extracted with the same volume of EtOAc three times (each 1 h) ultrasonically. The combined EtOAc extracts were concentrated to afford a crude extract (0.4 g), which was subjected to reversed-phase ODS column chromatography eluting with MeOH-H_2_O (from 40% to 100%) to afford 6 fractions (Fr.A–Fr.F). Fr.E (40 mg) was passed through a Sephadex LH-20 column (MeOH) and yielded mixtures of 5.0 mg of leucinostatins A and B. The structure of the mixtures was further identified by standard substance using LC-MS analysis. Approximately 200 mL of culture medium were used for comparative LC-MS analysis between PLBJ-1 and its mutants. LC-MS was performed on an Agilent Accurate-Mass-QTOF LC/MS 6520 instrument. HPLC analysis was performed on a Waters HPLC system (Waters e2695, Waters 2998, Photodiode Array Detector) using an ODS column (C18, 250 × 4.6 mm, YMC Pak, 5 μm). The ODS (50 μm) column was produced by YMC Co. Ltd. (Kyoto, Japan). The Sephadex LH-20 was purchased from GE Healthcare. Analytical HPLC was conducted with a Waters HPLC system (Waters e2695, Waters 2998, Photodiode Array Detector) using an ODS column (C18, 250 × 4.6 mm, YMC Pak, 5 μm) with a flow rate of 1 mL/min. The fresh extracts were dissolved in methanol before being separated on a linear gradient of MeOH:H_2_O (0.1% formic acid) at a flow rate of 1 mL/min. Fresh extracts of mutant strains were detected for 30 min using a linear gradient of 20% to 100% (0–20 min), 100% MeOH (20–25 min), and 20% MeOH (25–30 min). The LC-MS analysis method was consistent with analytical HPLC.

### Fungus bioassay

Confronting incubation of *P*. *lilacinum* (wild type, Δ*lcsA* and OE::*lcsF*) with *P*. *infestans* was performed on rye agar medium in 9 cm Petri plates, incubated simultaneously and cultured at 28°C for 24 h and then at 18°C, the optimum temperature for *P*. *infestans*, for 9 days, while confrontation with *P*. *capsici* was performed on lab-made PDA medium, cultured at 28°C for 7 days. For the inhibitory zone experiment, freshly produced sporangia of *P*. *infestans* was suspended in sterile water at a concentration of 2×10^5^ sporangia/mL. One milliliter of the suspension was smeared on 15 cm Petri plates, followed by Oxford cups with a diameter of 1 cm being placed. From 5 to 60 μg (increment 5 μg) of leucinostatins A and B dissolved in 20% methanol were added to the Oxford cup, and 20 μL of 20% methanol were used a control. Then, the Oxford cups were removed after the solution was absorbed by media. Five days later, the area was calculated by drawing circles of the inhibitory zone on metric graph paper and counting the number of square millimeters within the circle [110, 111]. Three biological replicates were performed. At the same time, the effects of 50 μg, 33 μg, 17 μg and 8.5 μg of leucinostatins are demonstrated in S11 Fig.

### Accession numbers

The genome sequences of PLBJ-1, PLFJ-1, and *P*. *chlamydosporium* strain 170 used for comparative analysis have been deposited at GenBank under the accession numbers LSBH00000000, LSBI00000000 and LSBJ00000000, respectively.

## Supporting Information

S1 Fig Genomic synteny of PLBJ-1 and TERIBC 1. Syntenic relationships were analyzed by BLASTN with an E-value cutoff of 1e-5. The red semicircle represents the scaffolds of PLBJ-1, while the blue semicircle represents the scaffolds of TERIBC 1. Scaffold lengths of ≥ 100 Kb were used for this analysis, and the threshold of the matched block was ≥ 1000 bp, which was connected by lines of the same color.
(TIF)

S2 Fig KOG classification of PLBJ-1 and PLFJ-1 genomes and proteins related to the PHI database. (TIF)

S3 Fig Maximum likelihood phylogeny of the PKSs in PLBJ-1 and other identified fungal PKSs. The domain structure of PKSs was predicted by Pfam and antiSMASH, and KS domains were used for phylogenetic analysis. Bootstraps values >50% are presented on the nodes.
(PDF)

S4 Fig Maximum likelihood phylogeny of the NRPS A domains from fungi in Ophiocordycipitaceae. The tree included NRPSs from Ophiocordycipitaceae (*P*. *liliacinum*, *T*. *inflatum*, *T*. *ophioglossoides*, *H*. *minnesotensis*, *O*. *sinensis* and *O*. *unilateralis* **)** and some functionally characterized products. The sub-clades of peptaibiotics, siderophore synthetase and cyclosporine synthetase are highlighted by shading.
(PDF)

S5 Fig Strategy and identification of the *lcsA* deletion. (A) Double homologous recombination strategy for deleting 2,613 bp of *lcsA* by introducing the homologous arm into the plasmid pKOV21. (B) Candidate transformants grown on medium resulted from either homologous recombination or ectopic integration of the *neo* gene cassette into the genome. (C) PCR amplification verified the validity of Δ*lcsA*. Lanes 1 and 2, amplified with primer pair targetF and targetR, identified the target gene that was deleted from *P*. *lilacinum*. Lanes 3 and 4 were amplified with primer pair upcheckF and neoupR, and lanes 5 and 6 were amplified with primer pair neodownF and upcheckR. The bonds in lanes 4 and 6 indicated the *neo* replaced the target gene.
(TIF)

S6 Fig MS profile of the extracts of the wild type and mutant *P*. *lilacinum* cultures. Peaks of [M+H]^+^ = 1219 (leucinostatin A) and [M+H]^+^ = 1205 (leucinostatin B) were detected in wild type, but not in the Δ*lcsA*, Δ*lcsC*, Δ*lcsD* and Δ*lcsE*.
(TIF)

S7 Fig The boundary of the *lcs* cluster identified by RNA-seq and qRT-PCR. (A) Expression levels in FPKM for genes in and flanking the *lcs* cluster in leucinostatin-inducing medium. (B) Expression ratio of genes in wild type PLBJ-1 to those in Δ*lcsA* when cultured in leucinostatin-inducing medium.
(TIF)

S8 Fig Identification of the Δ*lcsC*, Δ*lcsD* and Δ*lcsE* transformants and overexpression of transcription factor *lcsF*. (A) PCR amplification verified the validity of Δ*lcsC*, Δ*lcsD and* Δ*lcsE*. Lanes 1 and 2 were amplified for target gene products, lanes 3 and 4 were amplified with primer pairs in *neo* and upstream of the knockout cassette, and lanes 5 and 6 were amplified with primer pairs in *neo* and downstream of the knockout cassette. (B) Overexpression cassette of KSTNP-OE*lcsF* including PtrpC promoter, gene *lcsF* and TrpC terminator, using *neo* as the marker. (C) The approximate 3,000 bp fragments in lanes 2, 3 and 4 were amplified from three overexpressing transformants with the primer pair OE*lcsF*F and OE*lcsF*R, and lane 1 was amplified from wild type *P*. *lilacinum*.
(TIF)

S9 Fig *lcsF* overexpression increased the expression of related genes and leucinostatin production. (A) Overexpression of transcription factor *lcsF* enhanced expression levels of genes in the *lcs* cluster. The expression ratio referred to the genes in OE::*lcsF* to those in the wild type when cultured in leucinostatin-inducing medium. (B) HPLC profiles (UV 210 nm) of culture extracts from the wild type *P*. *lilacinum* strain and three overexpressing transformants cultured in PDB medium.
(TIF)

S10 Fig Cocultivation of *P*. *infestans* with *P*. *lilacinum* wild type, Δ*lcsC*, Δ*lcsD* and Δ*lcsE* mutants on rye agar medium. (TIF)

S11 Fig Inhibitory zone of *P*. *infestans* with leucinostatins A and B of different concentration. The location of leucinostatins was represented by black circle, of which the dosage is marked. The red dotted line circles the inhibitory zones of *P*. *infestans*. Twenty microliters of 20% methanol were placed at CK.
(TIF)

S12 Fig The area (mm^2^) of the inhibitory zone with different leucinostatin dosages. (TIF)

S13 Fig Syntenic relationships of *lcs* cluster in PLBJ-1, PLFJ-1 and TERIBC 1. Syntenic relationships were analyzed by BLASTN, with an E-value cutoff of 1e-5. The *lcs* cluster was located in scaffold00004 (1,048,997–1,162,497 bp) of PLFJ-1, scaffold00002 (1,030,100–1,143,599 bp) of PLBJ-1 and LOFA01000012.1 (1,012,032–1,125,446 bp) of TERIBC 1. The genes in this region are indicated by blue arrows.
(TIF)

S14 Fig Maximum likelihood phylogeny of the ten A domains of LcsA. (TIF)

S1 Table Genome size and repeat sequence in *P*. *lilacinum* and other fungi. (DOCX)

S2 Table Super scaffold construction, based on syntenic alignments of PLBJ-1 and PLFJ-1. (DOCX)

S3 Table Comparison of repeat elements between PLBJ-1 and PLFJ-1. (DOCX)

S4 Table Glycoside hydrolases in *P*. *lilacinum* and comparison with other fungi. (XLSX)

S5 Table Carbohydrate-binding modules in *P*. *lilacinum* and comparison with other fungi. (XLSX)

S6 Table Carbohydrate esterases in *P*. *lilacinum* and comparison with other fungi. (XLSX)

S7 Table Glycosyltransferases in *P*. *lilacinum* and comparison with other fungi. (XLSX)

S8 Table Proteases in *P*. *lilacinum* and comparison with other fungi. (XLSX)

S9 Table Paralogous expansion of some protein families in PLBJ-1 and PLFJ-1. (DOCX)

S10 Table Major paralogous gene expansion in nematode parasitic fungi. (DOCX)

S11 Table Biosynthetic gene clusters of secondary metabolites in *P*. *lilacinum*. (DOCX)

S12 Table A-domain selectivity of LcsA predicted by NRPSpredictor2. (DOCX)

S13 Table Primer sequences used in this study. (DOCX)

S14 Table Fungus strains used in the phylogenomic analysis. (DOCX)
