# Supplementary figures and images for: Biosynthesis of Antibiotic Leucinostatins in Bio-control Fungus Purpureocillium lilacinum and Their Inhibition on Phytophthora Revealed by Genome Mining
Source: PLoS Pathog. 2016 Jul 14;12(7):e1005685. doi: 10.1371/journal.ppat.1005685 (PMC4946873; doi:10.1371/journal.ppat.1005685)

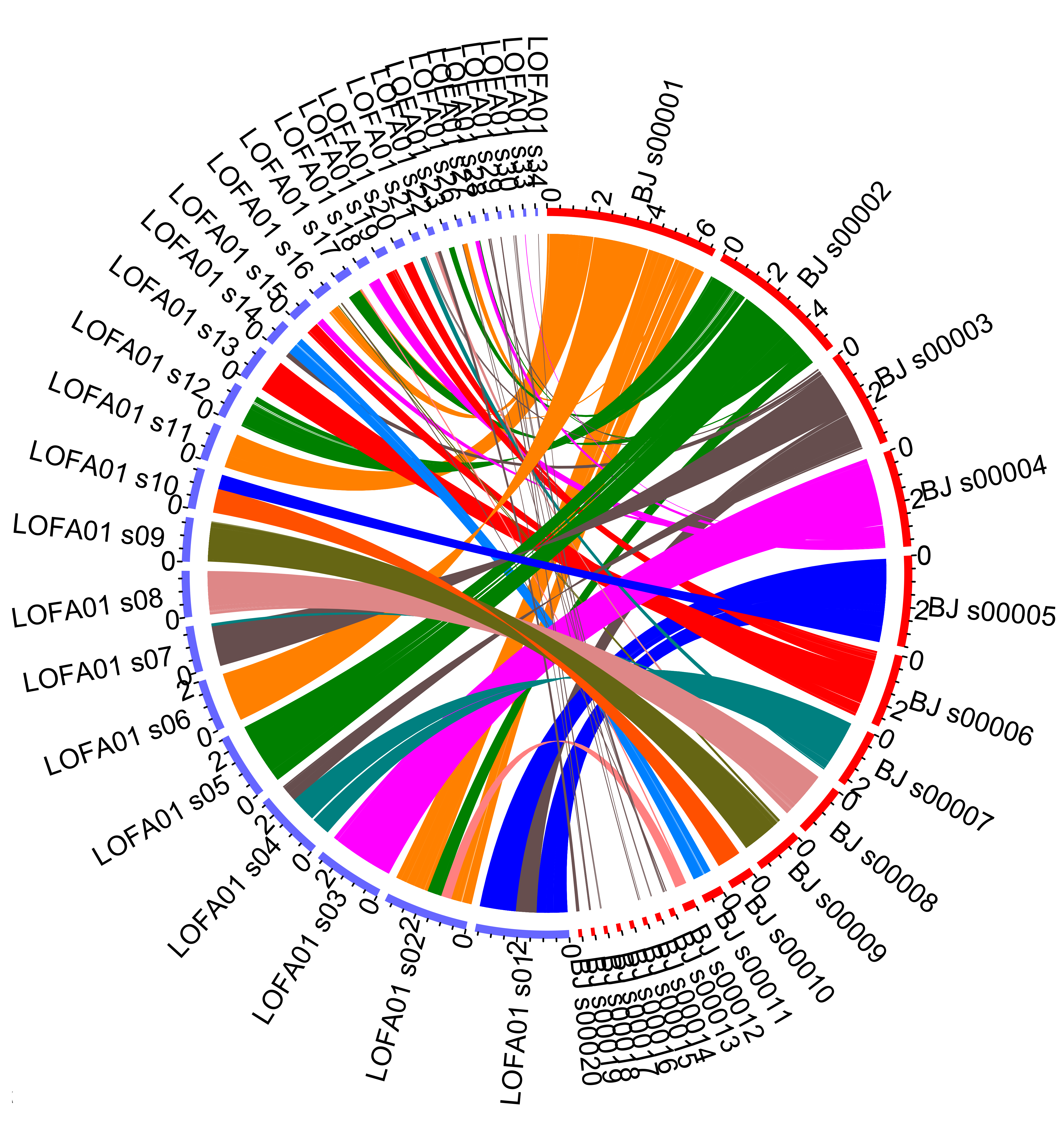

Supplement: S1 Fig — Syntenic relationships were analyzed by BLASTN with an E-value cutoff of 1e-5. The red semicircle represents the scaffolds of PLBJ-1, while the blue semicircle represents the scaffolds of TERIBC 1. Scaffold lengths of ≥ 100 Kb were used for this analysis, and the threshold of the matched block was ≥ 1000 bp, which was connected by lines of the same color. (TIF) [file ppat.1005685.s001.tif]

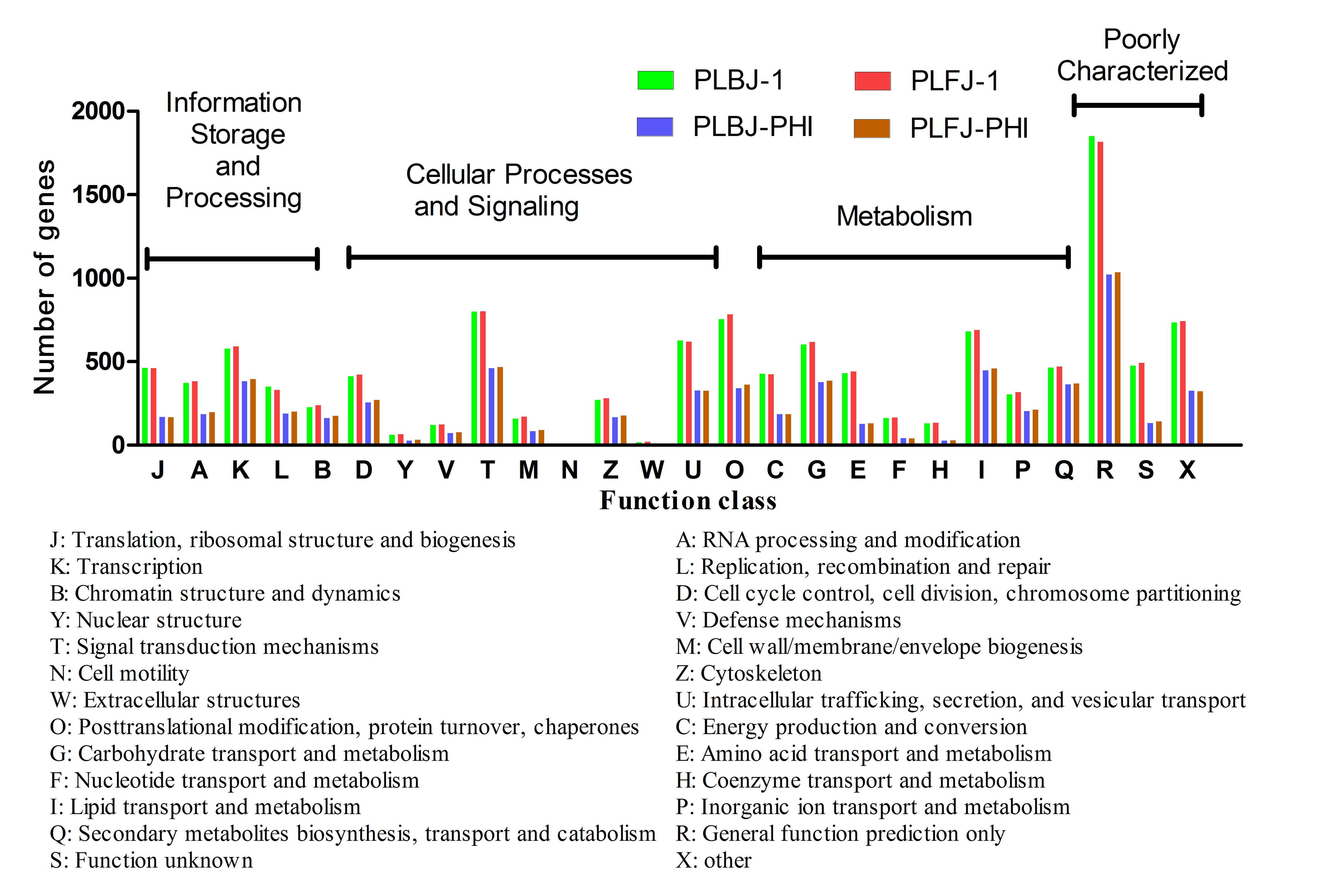

Supplement: S2 Fig — (TIF) [file ppat.1005685.s002.tif]

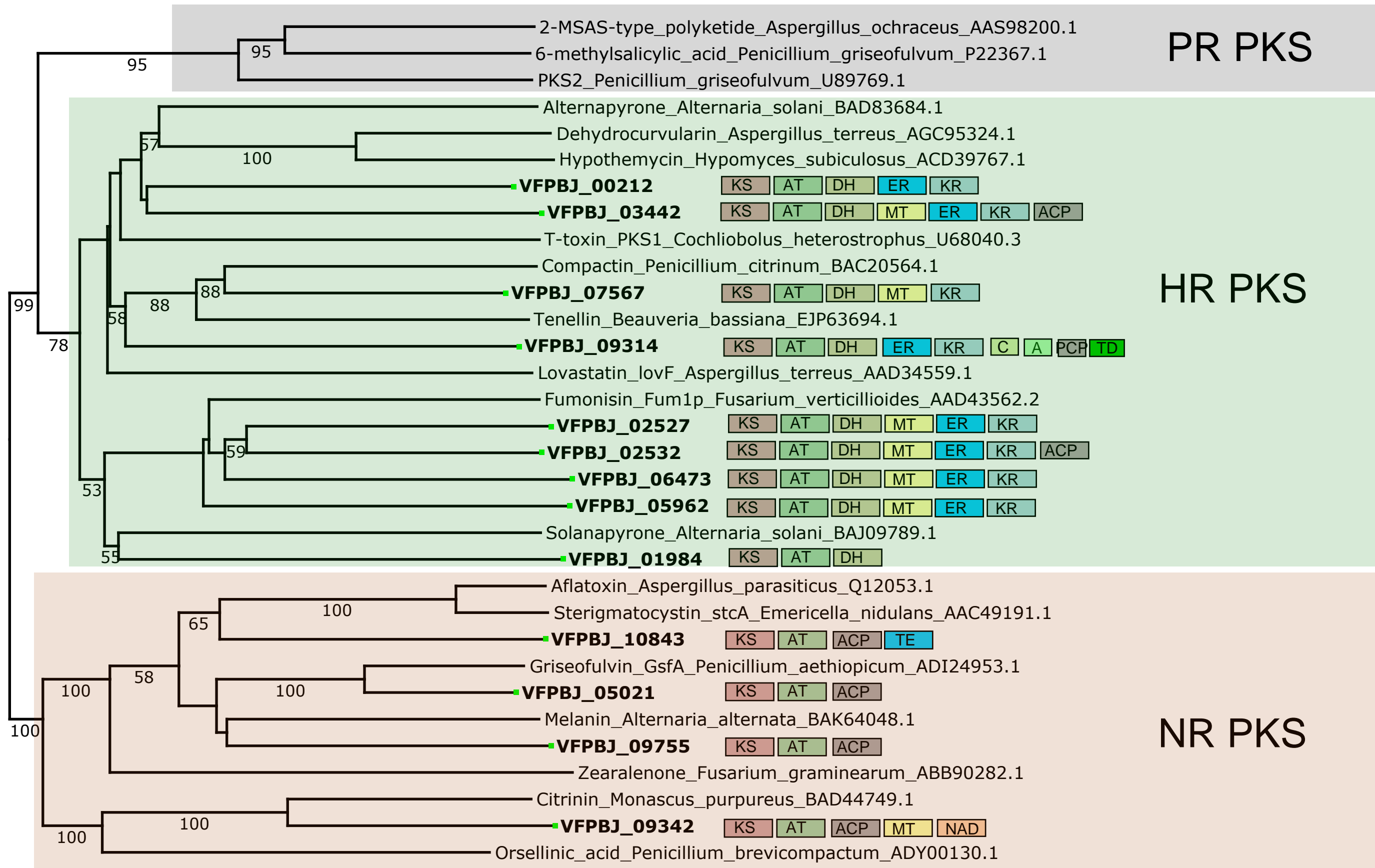

Supplement: S3 Fig — The domain structure of PKSs was predicted by Pfam and antiSMASH, and KS domains were used for phylogenetic analysis. Bootstraps values >50% are presented on the nodes. (PDF) [file ppat.1005685.s003.pdf]

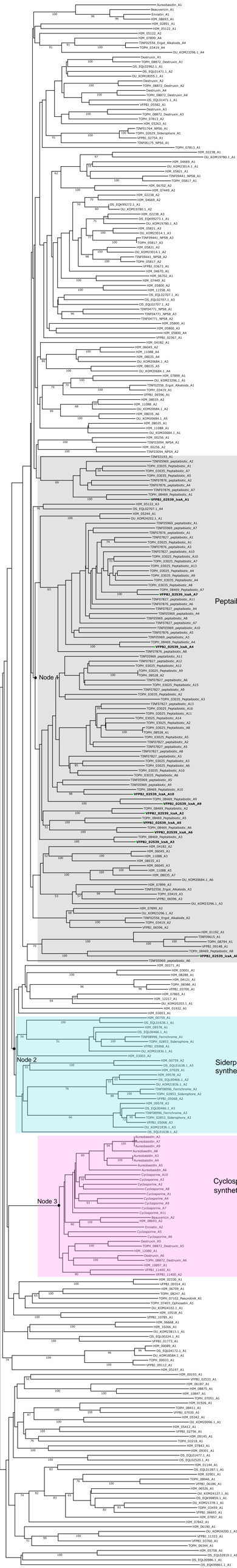

Supplement: S4 Fig — The tree included NRPSs from Ophiocordycipitaceae (P. liliacinum, T. inflatum, T. ophioglossoides, H. minnesotensis, O. sinensis and O. unilateralis ) and some functionally characterized products. The sub-clades of peptaibiotics, siderophore synthetase and cyclosporine synthetase are highlighted by shading. (PDF) [file ppat.1005685.s004.pdf]

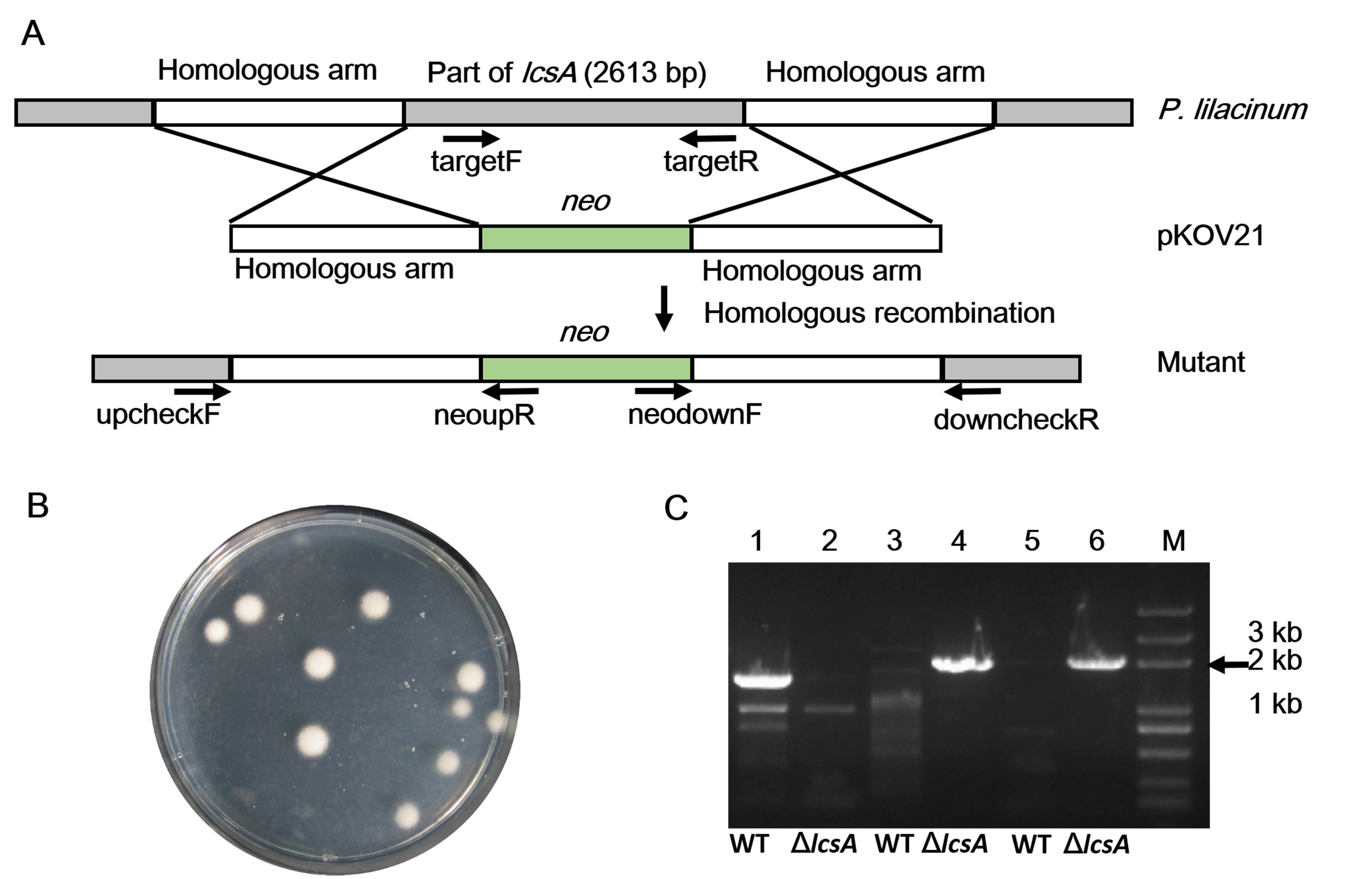

Supplement: S5 Fig — (A) Double homologous recombination strategy for deleting 2,613 bp of lcsA by introducing the homologous arm into the plasmid pKOV21. (B) Candidate transformants grown on medium resulted from either homologous recombination or ectopic integration of the neo gene cassette into the genome. (C) PCR amplification verified the validity of ΔlcsA. Lanes 1 and 2, amplified with primer pair targetF and targetR, identified the target gene that was deleted from P. lilacinum. Lanes 3 and 4 were amplified with primer pair upcheckF and neoupR, and lanes 5 and 6 were amplified with primer pair neodownF and upcheckR. The bonds in lanes 4 and 6 indicated the neo replaced the target gene. (TIF) [file ppat.1005685.s005.tif]

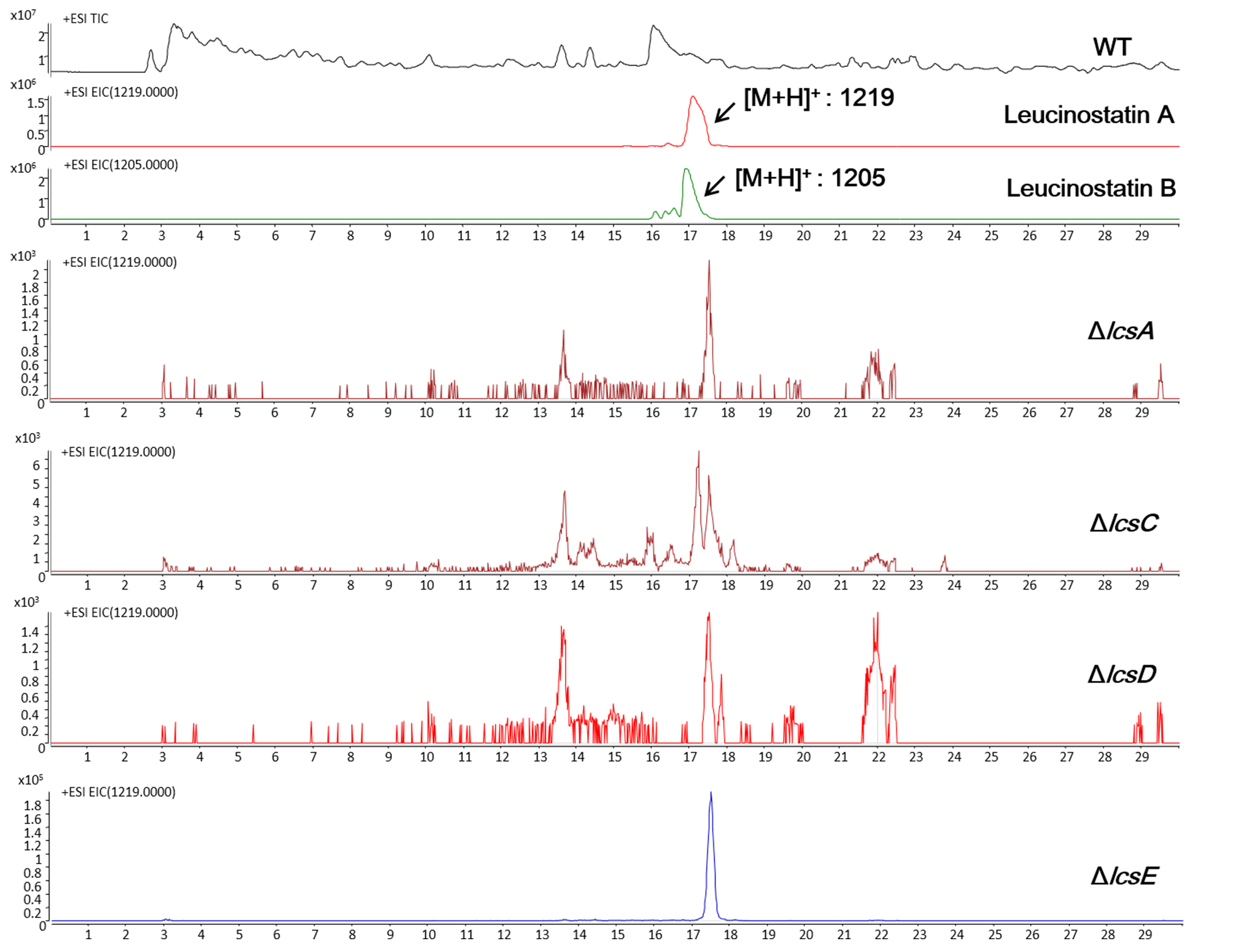

Supplement: S6 Fig — Peaks of [M+H]+ = 1219 (leucinostatin A) and [M+H]+ = 1205 (leucinostatin B) were detected in wild type, but not in the ΔlcsA, ΔlcsC, ΔlcsD and ΔlcsE. (TIF) [file ppat.1005685.s006.tif]

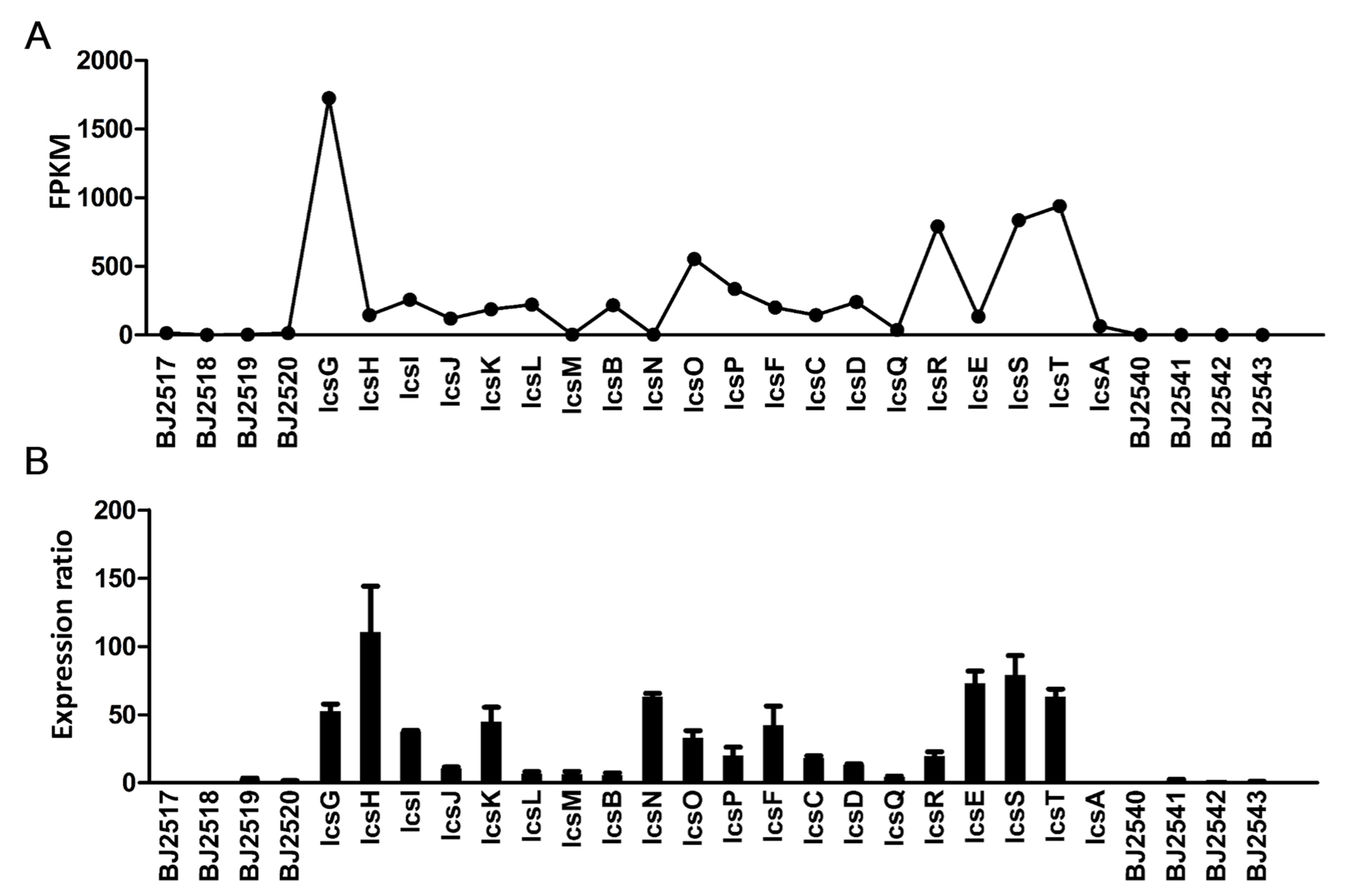

Supplement: S7 Fig — (A) Expression levels in FPKM for genes in and flanking the lcs cluster in leucinostatin-inducing medium. (B) Expression ratio of genes in wild type PLBJ-1 to those in ΔlcsA when cultured in leucinostatin-inducing medium. (TIF) [file ppat.1005685.s007.tif]

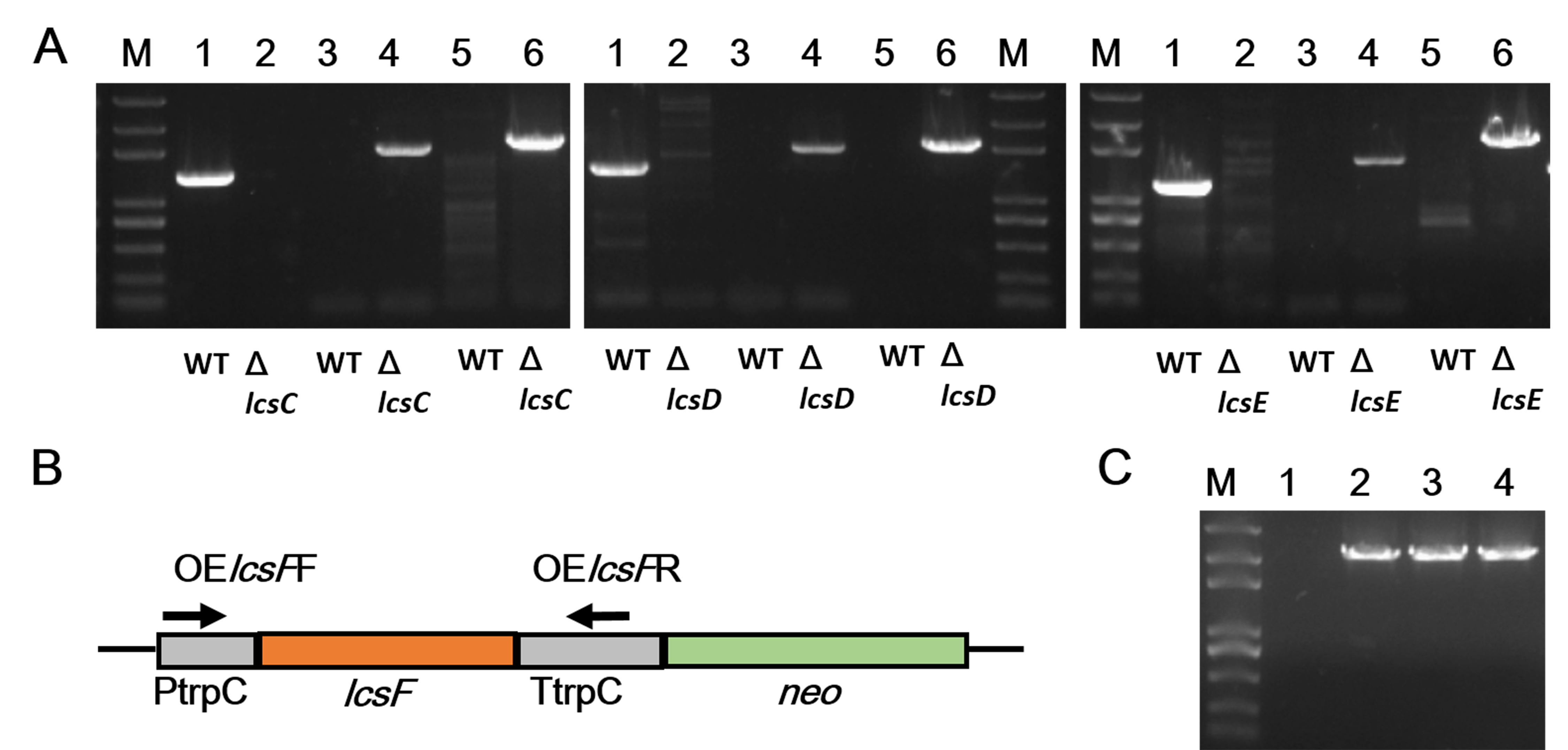

Supplement: S8 Fig — (A) PCR amplification verified the validity of ΔlcsC, ΔlcsD and ΔlcsE. Lanes 1 and 2 were amplified for target gene products, lanes 3 and 4 were amplified with primer pairs in neo and upstream of the knockout cassette, and lanes 5 and 6 were amplified with primer pairs in neo and downstream of the knockout cassette. (B) Overexpression cassette of KSTNP-OElcsF including PtrpC promoter, gene lcsF and TrpC terminator, using neo as the marker. (C) The approximate 3,000 bp fragments in lanes 2, 3 and 4 were amplified from three overexpressing transformants with the primer pair OElcsFF and OElcsFR, and lane 1 was amplified from wild type P. lilacinum. (TIF) [file ppat.1005685.s008.tif]

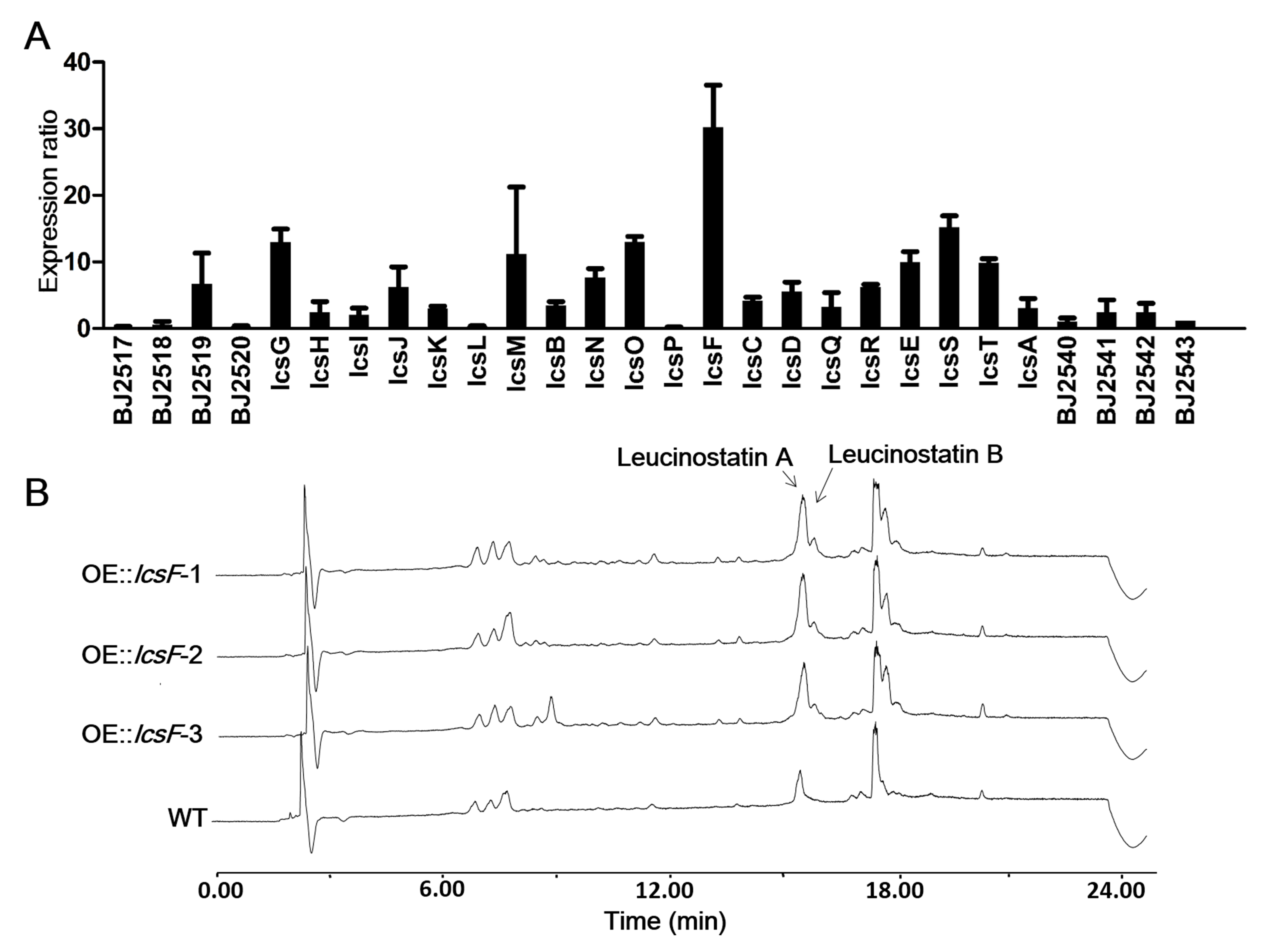

Supplement: S9 Fig — (A) Overexpression of transcription factor lcsF enhanced expression levels of genes in the lcs cluster. The expression ratio referred to the genes in OE::lcsF to those in the wild type when cultured in leucinostatin-inducing medium. (B) HPLC profiles (UV 210 nm) of culture extracts from the wild type P. lilacinum strain and three overexpressing transformants cultured in PDB medium. (TIF) [file ppat.1005685.s009.tif]

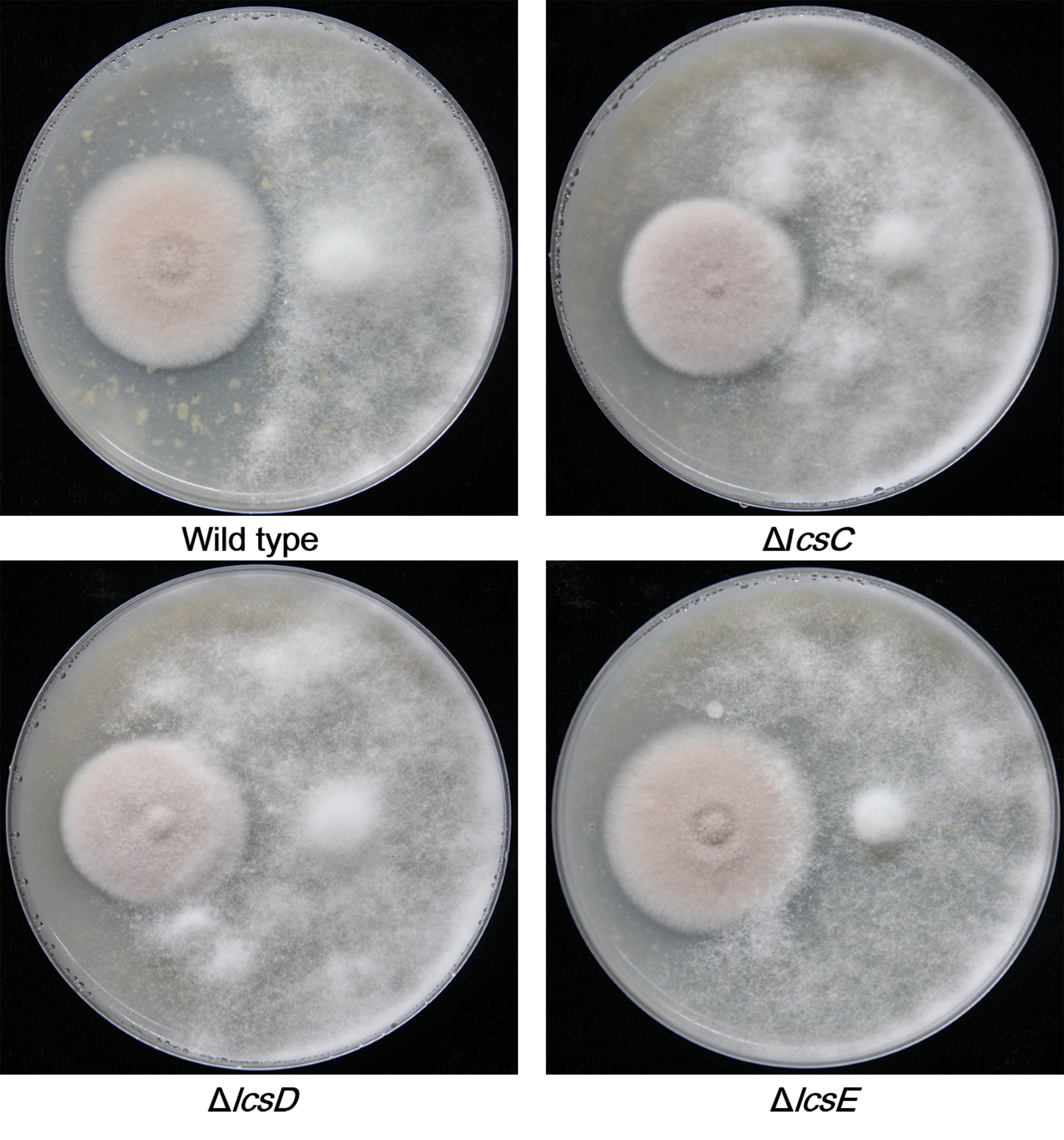

Supplement: S10 Fig — (TIF) [file ppat.1005685.s010.tif]

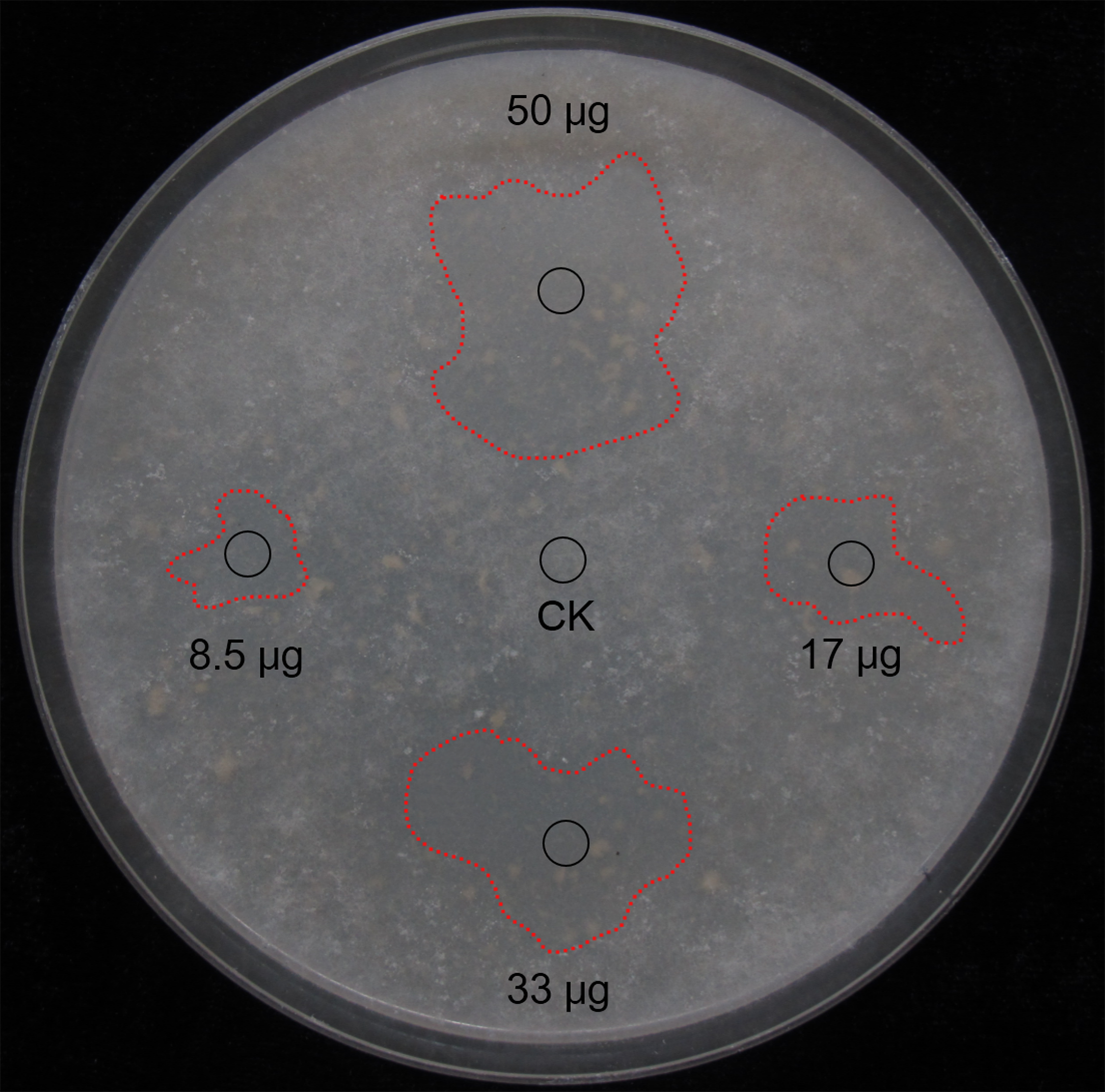

Supplement: S11 Fig — The location of leucinostatins was represented by black circle, of which the dosage is marked. The red dotted line circles the inhibitory zones of P. infestans. Twenty microliters of 20% methanol were placed at CK. (TIF) [file ppat.1005685.s011.tif]

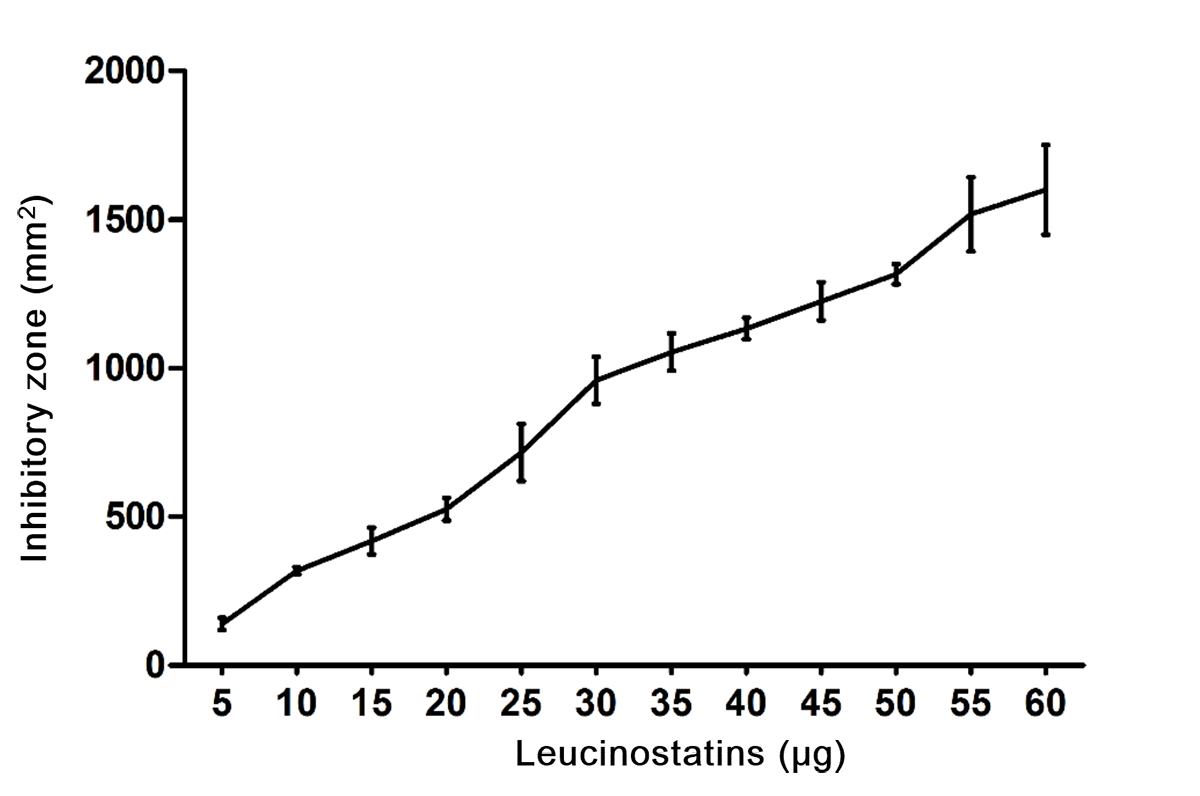

Supplement: S12 Fig — (TIF) [file ppat.1005685.s012.tif]

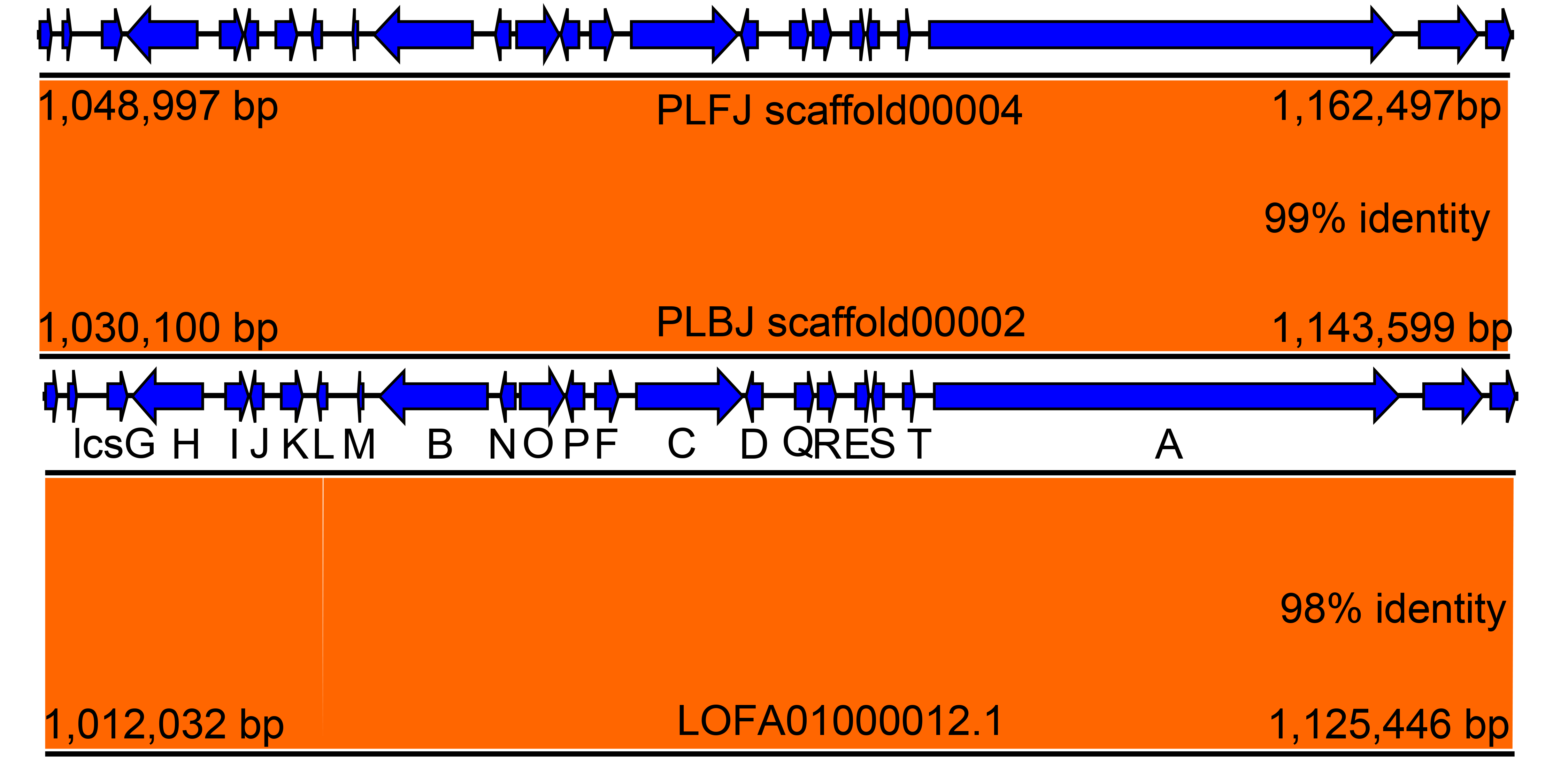

Supplement: S13 Fig — Syntenic relationships were analyzed by BLASTN, with an E-value cutoff of 1e-5. The lcs cluster was located in scaffold00004 (1,048,997–1,162,497 bp) of PLFJ-1, scaffold00002 (1,030,100–1,143,599 bp) of PLBJ-1 and LOFA01000012.1 (1,012,032–1,125,446 bp) of TERIBC 1. The genes in this region are indicated by blue arrows. (TIF) [file ppat.1005685.s013.tif]
