# Supplementary material for: Biosynthesis of Antibiotic Leucinostatins in Bio-control Fungus Purpureocillium lilacinum and Their Inhibition on Phytophthora Revealed by Genome Mining
Source: PLoS Pathog. 2016 Jul 14;12(7):e1005685. doi: 10.1371/journal.ppat.1005685 (PMC4946873; doi:10.1371/journal.ppat.1005685)
Supplement: S1 Table — (DOCX) [file ppat.1005685.s015.docx]

**Table S1 Genome size and repeat sequence in *P. lilacinum* and other fungi.**

|  | PLBJ | PLFJ | P170 | HMI | BBA | CMI | MRO | TIN | OSI | TOP | TRE | FOX | AOL | MHA |
| --- | --- | --- | --- | --- | --- | --- | --- | --- | --- | --- | --- | --- | --- | --- |
| Size (Mb) | 38.1 | 38.5 | 43.9 | 51.4 | 33.7 | 32.2 | 39.0 | 30.5 | 120 | 31.2 | 34.1 | 60.0 | 40.1 | 40.4 |
| Repeat  (%) | 6.07 | 6.00 | 5.24 | 34.67 | 2.03 | 3.04 | 0.98 | 1.24 | 37.98 | -- | 2.11 | 28 | 0.47 | 3.8 |
| Genes | 11773 | 11763 | 14890 | 12702 | 10366 | 9684 | 10582 | 9998 | 6972 | 10134 | 9129 | 17735 | 11479 | 10959 |

PLBJ, *Purpureocillium lilacinum* strain PLBJ-1; PLFJ, *Purpureocillium lilacinum* strain PLFJ-1; P170, *Pochonia chlamydosporia* strain 170; HMI, *Hirsutella minnesotensis*; BBA, *Beauveria bassiana*; CMI, *Cordyceps militaris*; MRO, *Metarhizium robertsii*; TIN, *Tolypocladium inflatum*; OSI, *Ophiocordyceps sinensis*; TOP, *Tolypocladium ophioglossoides*; TRE, *Trichoderma reesei*; FOX, *Fusarium oxysporum* (*Fol*); AOL, *Arthrobotrys oligospora*; MHA, *Monacrosporium haptotylum.*
