# Supplementary material for: Biosynthesis of Antibiotic Leucinostatins in Bio-control Fungus Purpureocillium lilacinum and Their Inhibition on Phytophthora Revealed by Genome Mining
Source: PLoS Pathog. 2016 Jul 14;12(7):e1005685. doi: 10.1371/journal.ppat.1005685 (PMC4946873; doi:10.1371/journal.ppat.1005685)
Supplement: S2 Table — (DOCX) [file ppat.1005685.s016.docx]

**Table S2 Super scaffold construction, based on syntenic alignments of PLBJ-1 and PLFJ-1.**

| Super scaffold ID | FLBJ-1 | strand | PLFJ-1 | Strand |
| --- | --- | --- | --- | --- |
| super scaffold1 | scaffold00001 | + | scaffold00001 | + |
|  |  |  | scaffold00012 | + |
| super scaffold2 | scaffold00002 | + | scaffold00004 | + |
|  | scaffold00012 | + | scaffold00005 | + |
| super scaffold3 | scaffold00010 | + | scaffold00002 | + |
|  | scaffold00005 | + |  |  |
| super scaffold4 | scaffold00005 | + | scaffold00024 | + |
|  |  |  | scaffold00013 | + |
| super scaffold5 | scaffold00011 | + | scaffold00014 | + |
|  | scaffold00003 | + | scaffold00022 | + |
|  |  |  | scaffold00003 | + |
| super scaffold6 | scaffold00018 | + | scaffold00023 | + |
|  |  |  | scaffold00021 | + |
| super scaffold7 | scaffold00004 | + | scaffold00006 | - |
|  |  |  | scaffold00016 | - |
|  |  |  | scaffold00015 | + |
| super scaffold8 | scaffold00006 | + | scaffold00027 | + |
|  |  |  | scaffold00007 | - |
| super scaffold9 | scaffold00009 | + | scaffold00008 | + |
|  | scaffold00007 | + | scaffold00010 | + |
|  |  |  | scaffold00011 | + |
|  |  |  | scaffold00020 | + |
| super scaffold10 | scaffold00008 | + | scaffold00009 | + |
|  |  |  | scaffold00017 | - |
