# Supplementary material for: Biosynthesis of Antibiotic Leucinostatins in Bio-control Fungus Purpureocillium lilacinum and Their Inhibition on Phytophthora Revealed by Genome Mining
Source: PLoS Pathog. 2016 Jul 14;12(7):e1005685. doi: 10.1371/journal.ppat.1005685 (PMC4946873; doi:10.1371/journal.ppat.1005685)
Supplement: S3 Table — (DOCX) [file ppat.1005685.s017.docx]

**Table S3 Comparison of repeat elements between PLBJ-1 and PLFJ-1.**

|  |  |  | PLBJ | | | PLFJ | | |
| --- | --- | --- | --- | --- | --- | --- | --- | --- |
| Class | Type | | Length (bp) | Per  (%) | Number | Length (bp) | Per  (%) | Number |
| ClassI | DIRS | DIRS | 26161 | 0.07 | 46 | 17983 | 0.05 | 29 |
| ClassI | LINE | I | 432273 | 1.13 | 615 | 308628 | 0.80 | 423 |
| ClassI | LINE | Jockey | 4662 | 0.01 | 26 | 15961 | 0.04 | 136 |
| ClassI | LINE | L1 | 1071 | 0.00 | 8 | 8189 | 0.02 | 21 |
| ClassI | LINE | R2 | 328 | 0.00 | 2 | 4141 | 0.01 | 12 |
| ClassI | LINE | RTE | 1592 | 0.00 | 14 | 125 | 0.00 | 1 |
| ClassI | LTR | Bel-Pao | 2161 | 0.01 | 15 | 424 | 0.00 | 6 |
| ClassI | LTR | Copia | 122143 | 0.32 | 223 | 77375 | 0.20 | 211 |
| ClassI | LTR | ERV | 21530 | 0.06 | 42 | 944 | 0.00 | 4 |
| ClassI | LTR | Gypsy | 62953 | 0.17 | 119 | 114575 | 0.30 | 164 |
| ClassI | LTR | Retrovirus | 107 | 0.00 | 1 | 0 | 0.00 | 0 |
| ClassI | LTR | unknown | 1597 | 0.00 | 5 | 617 | 0.00 | 5 |
| ClassI | PLE | Penelope | 8879 | 0.02 | 22 | 0 | 0.00 | 0 |
| ClassI | SINE | tRNA | 293 | 0.00 | 4 | 293 | 0.00 | 4 |
| ClassI | SINE | unknown | 161 | 0.00 | 2 | 148 | 0.00 | 2 |
| ClassII | Crypton | Crypton | 1259 | 0.00 | 6 | 1624 | 0.00 | 7 |
| ClassII | Helitron | Helitron | 27835 | 0.07 | 47 | 18455 | 0.05 | 52 |
| ClassII | Maverick | Maverick | 146 | 0.00 | 2 | 51 | 0.00 | 1 |
| ClassII | TIR | CACTA | 9342 | 0.02 | 36 | 4658 | 0.01 | 29 |
| ClassII | TIR | PIF-Harbinger | 5166 | 0.01 | 24 | 469 | 0.00 | 5 |
| ClassII | TIR | PiggyBac | 115 | 0.00 | 1 | 115 | 0.00 | 1 |
| ClassII | TIR | Tc1-Mariner | 13086 | 0.03 | 35 | 58408 | 0.15 | 91 |
| ClassII | TIR | hAT | 107593 | 0.28 | 253 | 10444 | 0.03 | 61 |
| ClassII | unknown | Ginger1 | 0 | 0.00 | 0 | 99 | 0.00 | 1 |
| ClassII | unknown | MuDR | 125280 | 0.33 | 234 | 62082 | 0.16 | 116 |
| ClassII | unknown | Novosib | 116 | 0.00 | 1 | 116 | 0.00 | 1 |
| ClassII | unknown | Sola | 821 | 0.00 | 5 | 542 | 0.00 | 3 |
| otherTE | unknown | unknown | 689553 | 1.81 | 2619 | 961332 | 2.49 | 2840 |
| Low  complexity | Low  complexity | Low  complexity | 45611 | 0.12 | 939 | 48877 | 0.13 | 996 |
| Simple  repeat | Simple  repeat | Simple  repeat | 604416 | 1.58 | 14550 | 596818 | 1.55 | 14345 |
| Sum | | | 2316250 | 6.07 | 19896 | 2313493 | 6.00 | 19567 |
