# Supplementary material for: Biosynthesis of Antibiotic Leucinostatins in Bio-control Fungus Purpureocillium lilacinum and Their Inhibition on Phytophthora Revealed by Genome Mining
Source: PLoS Pathog. 2016 Jul 14;12(7):e1005685. doi: 10.1371/journal.ppat.1005685 (PMC4946873; doi:10.1371/journal.ppat.1005685)
Supplement: S9 Table — (DOCX) [file ppat.1005685.s023.docx]

**Table S9 Paralogous expansion of some protein families in PLBJ-1 and PLFJ-1.**

| Cluster | Annotation | PLBJ-1 | PLFJ-1 | Other |
| --- | --- | --- | --- | --- |
| 1 | Reverse transcriptase | 40 | 30 | 10 |
| 2 | Reverse transcriptase | 21 | 15 | 9.4 |
| 3 | Oligopeptide transporter protein | 14 | 14 | 9.3 |
| 4 | MULE transposase | 21 | 7 | 4.1 |
| 5 | ABC transporter | 10 | 10 | 7.3 |
| 6 | Integrase core domain | 12 | 1 | 0.8 |
| 7 | Fn3-like domain | 7 | 8 | 2.7 |
| 8 | Cerato-platanin | 3 | 5 | 1.2 |
| 9 | hAT family | 3 | 8 | 3.1 |
| 10 | Pectate lyase superfamily | 7 | 7 | 3.8 |

These results were from OrthoMCL analysis of two *P. lilacinum* isolates PLBJ-1 and PLFJ-1 and 13 other fungi including *P. chlamydosporia* strains 123 and 170, *H. minnesotensis*, *B. bassiana*, *C. militaris*, *M. robertsii*, *T. inflatum*, *O. sinensis, T. reesei*, *T. ophioglossoides*, *F. oxysporum*, *A. oligospora* and *M. haptotylum.* The “Other” was the mean value among the 13 fungi. Annotation of clusters was based on Pfam and KOG classification.
