# Supplementary material for: Biosynthesis of Antibiotic Leucinostatins in Bio-control Fungus Purpureocillium lilacinum and Their Inhibition on Phytophthora Revealed by Genome Mining
Source: PLoS Pathog. 2016 Jul 14;12(7):e1005685. doi: 10.1371/journal.ppat.1005685 (PMC4946873; doi:10.1371/journal.ppat.1005685)
Supplement: S10 Table — (DOCX) [file ppat.1005685.s024.docx]

**Table S10 Major paralogous gene expansion in nematode parasitic fungi.**

|  |  | PLBJ-1 | PLFJ-1 | P170 | P123 | HMI | Other |
| --- | --- | --- | --- | --- | --- | --- | --- |
| 1 | Reverse transcriptase | 40 | 30 | 19 | 4 | 71 | 3.6 |
| 2 | MULE transposase | 21 | 7 | 16 | 2 | 17 | 1.8 |
| 3 | oligopeptide transporter protein | 14 | 14 | 14 | 15 | 9 | 8.3 |
| 4 | hAT family | 6 | 1 | 6 | 1 | 19 | 0.6 |
| 5 | Fn3-like domain | 7 | 8 | 7 | 4 | 3 | 2.1 |
| 6 | NO | 4 | 1 | 1 | 1 | 1 | 0.3 |
| 7 | hAT family | 4 | 1 | 6 | 3 | 18 | 0.5 |
| 8 | Major Facilitator Superfamily | 6 | 5 | 5 | 5 | 3 | 2.8 |
| 9 | F-box-like | 4 | 1 | 2 | 1 | 1 | 0.9 |
| 10 | ABC transporter | 10 | 10 | 9 | 10 | 7 | 6.9 |
| 11 | HAMP domain | 4 | 4 | 3 | 4 | 2 | 1 |
| 12 | Histidine kinase | 3 | 3 | 1 | 2 | 4 | 0.7 |
| 13 | hAT family | 3 | 8 | 8 | 1 | 23 | 0.8 |
| 14 | Fungal Zn(2)-Cys(6) binuclear cluster domain | 3 | 4 | 2 | 2 | 12 | 0.8 |
| 15 | MULE transposase domain | 3 | 2 | 6 | 1 | 18 | 0.8 |
| 16 | AMP-binding enzyme | 9 | 9 | 12 | 13 | 23 | 7 |
| 17 | Glycosyl hydrolase family | 4 | 4 | 7 | 7 | 3 | 2.2 |
| 18 | ABC transporter transmembrane region | 8 | 8 | 9 | 10 | 9 | 6.2 |
| 19 | PIF1-like helicase | 6 | 6 | 13 | 6 | 83 | 4.4 |
| 20 | Cytochrome P450 | 2 | 2 | 2 | 2 | 5 | 0.5 |
| 21 | Major Facilitator Superfamily | 3 | 4 | 5 | 5 | 2 | 1.6 |
| 22 | Carboxylesterase | 3 | 3 | 4 | 3 | 7 | 1.6 |
| 23 | alpha/beta hydrolase fold | 2 | 2 | 3 | 3 | 4 | 0.7 |
| 24 | Glycosyl hydrolase | 3 | 3 | 4 | 3 | 5 | 1.7 |
| 25 | short chain dehydrogenase | 2 | 2 | 1 | 1 | 4 | 0.7 |
| 26 | Glycosyl hydrolase | 4 | 4 | 6 | 6 | 3 | 2.9 |
| 27 | Cation transport protein | 2 | 2 | 4 | 3 | 3 | 0.9 |
| 28 | Peroxidase | 1 | 1 | 3 | 2 | 4 | 0.3 |
| 29 | FAD binding domain | 2 | 2 | 2 | 2 | 6 | 1.3 |
| 30 | Bacterial low temperature requirement A protein | 2 | 3 | 6 | 3 | 7 | 1.4 |
| 31 | bZIP transcription factor | 1 | 1 | 2 | 1 | 4 | 0.4 |
| 32 | Pregnancy-associated plasma protein-A | 1 | 1 | 1 | 1 | 4 | 0.4 |
| 33 | unknown function | 1 | 1 | 1 | 1 | 4 | 0.4 |
| 34 | AMP-binding enzyme | 2 | 2 | 2 | 2 | 5 | 1.5 |
| 35 | Acyl transferase domain | 3 | 3 | 5 | 5 | 9 | 2.6 |
| 36 | Cytochrome P450 | 2 | 2 | 5 | 5 | 6 | 1.7 |
| 37 | FAD dependent oxidoreductase | 1 | 1 | 2 | 2 | 4 | 0.7 |
| 38 | FAD binding domain | 1 | 2 | 1 | 1 | 4 | 0.8 |
| 39 | Cytochrome P450 | 2 | 2 | 5 | 5 | 3 | 1.8 |
| 40 | Fungal Zn(2)-Cys(6) binuclear cluster domain | 1 | 1 | 3 | 4 | 1 | 0.8 |
| 41 | alpha/beta hydrolase fold | 1 | 1 | 2 | 2 | 6 | 0.8 |
| 42 | Major Facilitator Superfamily | 1 | 1 | 2 | 2 | 4 | 0.8 |
| 43 | Mpv17 / PMP22 family | 1 | 1 | 1 | 1 | 4 | 0.8 |
| 44 | RNA polymerase Rpb1 | 2 | 2 | 3 | 5 | 2 | 1.9 |
| 45 | Mini-chromosome maintenance protein | 1 | 1 | 1 | 4 | 1 | 0.9 |
| 46 | unknown function | 1 | 1 | 1 | 1 | 6 | 0.9 |
| 47 | Phosphotransferase enzyme | 1 | 1 | 1 | 1 | 4 | 0.9 |
| 48 | Cytochrome P450 | 1 | 1 | 1 | 1 | 4 | 0.9 |

These results were from OrthoMCL analysis of two *P. lilacinum* isolates (PLBJ-1 and PLFJ-1), two *P. chlamydosporia* isolates (strain 170 and 123), HMI (*H. minnesotensis*) and ten other fungi including *B. bassiana*, *C. militaris*, *M. robertsii*, *T. inflatum*, *O. sinensis, T. reesei*, *T. ophioglossoides*, *F. oxysporum*, *A. oligospora* and *M. haptotylum.* The “Other” was the mean value among the ten fungi. Annotation of protein families was based on Pfam and KOG classification. The 48 selected protein families were all expand in the five nematode-related strains and at least one strain with three or more gene expansion against the “Other”.
