# Supplementary material for: Biosynthesis of Antibiotic Leucinostatins in Bio-control Fungus Purpureocillium lilacinum and Their Inhibition on Phytophthora Revealed by Genome Mining
Source: PLoS Pathog. 2016 Jul 14;12(7):e1005685. doi: 10.1371/journal.ppat.1005685 (PMC4946873; doi:10.1371/journal.ppat.1005685)
Supplement: S11 Table — (DOCX) [file ppat.1005685.s025.docx]

**Table S11 Biosynthetic gene clusters of secondary metabolites in *P. lilacinum*.**

| Gene ID | Type | FPKM | Domain structure | Cluster (antiSMASH) | Homolog in PLFJ-1 |
| --- | --- | --- | --- | --- | --- |
| VFPBJ_02367 | NRPS | 2.85 | PCP-C-PCP-C-A-PCP-C | VFPBJ_02360 ~ VFPBJ_02373 | VFPFJ_04539 |
| VFPBJ_02539 | NRPS | 65.39 | PCP-(C-A-PCP)*10-R | VFPBJ_02521 ~ VFPBJ_02546 | VFPFJ_04711 |
| VFPBJ_02754 | NRPS | 6.47 | A-PCP-C-PCP-C | VFPBJ_02748 ~ VFPBJ_02763 | VFPFJ_04930 |
| VFPBJ_03673 | NRPS | 0.70 | E-C-A-PCP | VFPBJ_03669 ~ VFPBJ_03678 | VFPFJ_05864 |
| VFPBJ_05068 | NRPS | 18.95 | A-PCP-C-A-PCP-C-PCP-C-A-C-PCP-C-C | VFPBJ_05064 ~ VFPBJ_05075 | VFPFJ_04271 |
| VFPBJ_06596 | NRPS | 0.16 | A-PCP-C-A-PCP-C-A | VFPBJ_06590 ~ VFPBJ_06602 | VFPFJ_02952 |
| VFPBJ_07030 | NRPS | 2.12 | A | VFPBJ_07021 ~ VFPBJ_07036 | VFPFJ_02509 |
| VFPBJ_09148 | NRPS | 4.00 | C-A-PCP-C | VFPBJ_09139 ~ VFPBJ_09154 | VFPFJ_09256 |
| VFPBJ_10785 | NRPS | 0 | C-A-PCP-C | VFPBJ_10776 ~ VFPBJ_10792 | VFPFJ_11097 |
| VFPBJ_11400 | NRPS | 0 | C-A-nMT-P-C-A | VFPBJ_11400 ~ VFPBJ_11402 | VFPFJ_09663 |
| VFPBJ_00212 | T1-PKS | 0.48 | KS-AT-DH-ER-KR | VFPBJ_00204 ~ VFPBJ_00217 | VFPFJ_00238 |
| VFPBJ_01984 | T1-PKS | 156.5 | KS-AT-DH | VFPBJ_01978 ~ VFPBJ_01991 | VFPFJ_06411 |
| VFPBJ_02527 | T1-PKS | 217.9 | KS-AT-DH-MT-ER-KR | VFPBJ_02521 ~ VFPBJ_02546 | VFPFJ_04699 |
| VFPBJ_02532 | T1-PKS | 145.6 | KS-AT-DH-MT-ER-KR-ACP | VFPBJ_02521 ~ VFPBJ_02546 | VFPFJ_04704 |
| VFPBJ_03442 | T1-PKS | 5.47 | KS-AT-DH-MT-ER-KR-ACP | VFPBJ_03436 ~ VFPBJ_03451 | VFPFJ_05625 |
| VFPBJ_05021 | T1-PKS | 0.08 | KS-AT-ACP | VFPBJ_05015 ~ VFPBJ_05027 | VFPFJ_04219 |
| VFPBJ_05962 | T1-PKS | 7.84 | KS-AT-DH-MT-ER-KR | VFPBJ_05958 ~ VFPBJ_05969 | VFPFJ_06681 |
| VFPBJ_06473 | T1-PKS | 398.4 | KS-AT-DH-MT-ER-KR | VFPBJ_06465 ~ VFPBJ_06483 | VFPFJ_10484 |
| VFPBJ_07567 | T1-PKS | 0.14 | KS-AT-DH-MT-KR | VFPBJ_07564 ~ VFPBJ_07576 | VFPFJ_08284 |
| VFPBJ_09342 | T1-PKS | 10.7 | KS-AT-ACP-MT-NAD | VFPBJ_09337 ~ VFPBJ_09349 | VFPFJ_09451 |
| VFPBJ_09755 | T1-PKS | 0.12 | KS-AT-ACP | VFPBJ_09747 ~ VFPBJ_09765 | VFPFJ_08369 |
| VFPBJ_10843 | T1-PKS | 0 | KS-AT-ACP-TE | VFPBJ_10834 ~ VFPBJ_10850 | VFPFJ_11160 |
| VFPBJ_08290 | T3-PKS | 0.16 |  | VFPBJ_08283 ~ VFPBJ_08297 | VFPFJ_07564 |
| VFPBJ_09314 | PKS-NRPS | 1.08 | KS-AT-DH-ER-KR-C-A-PCP-TD | VFPBJ_09311 ~ VFPBJ_09323 | VFPFJ_09423 |
| VFPBJ_01773 | NRPS-Like | 0.34 |  | VFPBJ_01766 ~ VFPBJ_01779 | VFPFJ_10289 |
| VFPBJ_03700 | NRPS-Like | 13.4 |  | VFPBJ_03694 ~ VFPBJ_03707 | VFPFJ_05892 |
| VFPBJ_03760 | NRPS-Like | 0.14 |  | VFPBJ_03751 ~ VFPBJ_03767 | VFPFJ_05952 |
| VFPBJ_04166 | NRPS-Like | 74.6 |  | VFPBJ_04166 ~ VFPBJ_04169 | VFPFJ_03378 |
| VFPBJ_05582 | NRPS-Like | 1.49 |  | VFPBJ_05575 ~ VFPBJ_05587 | VFPFJ_07063 |
| VFPBJ_06186 | NRPS-Like | 3.15 |  | VFPBJ_06177 ~ VFPBJ_06191 | VFPFJ_10737 |
| VFPBJ_06693 | NRPS-Like | 2.14 |  | VFPBJ_06684 ~ VFPBJ_06701 | VFPFJ_02850 |
| VFPBJ_09112 | NRPS-Like | 32.2 |  | VFPBJ_09103 ~ VFPBJ_09119 | VFPFJ_09219 |
| VFPBJ_10293 | NRPS-Like | 12.7 |  | VFPBJ_10284 ~ VFPBJ_10295 | VFPFJ_08916 |
| VFPBJ_11333 | NRPS-Like | 1.19 |  | VFPBJ_11329 ~ VFPBJ_11341 | VFPFJ_10843 |
| VFPBJ_01364 | PKS-Like | 0.17 | AT-KR-PCP-TD | VFPBJ_01354 ~ VFPBJ_01373 | VFPFJ_01384 |
| VFPBJ_03512 | PKS-Like | 8.75 |  | VFPBJ_03512 ~ VFPBJ_03519 | VFPFJ_05698 |
| VFPBJ_05217 | DMATS | 16.6 |  | VFPBJ_05212 ~ VFPBJ_05220 | VFPFJ_07433 |
| VFPBJ_06180 | TS | 0.63 |  | VFPBJ_06177 ~ VFPBJ_06191 | VFPFJ_10743 |
| VFPBJ_08301 | TS | 3.94 |  | VFPBJ_08297 ~ VFPBJ_08305 | VFPFJ_07553 |
| VFPBJ_08809 | TS | 49.7 |  | VFPBJ_08805 ~ VFPBJ_08811 | VFPFJ_10019 |
| VFPBJ_09911 | TS | 42.9 |  | VFPBJ_09909 ~ VFPBJ_09916 | VFPFJ_08522 |

Gene clusters involved in the secondary metabolite biosynthesis in PLBJ-1 and PLFJ-1 were predicted by antiSMASH and SMURF; Domain structures and gene clusters were identified by antiSMASH and Pfam; Core genes expression levels were demonstrated by FPKM value when PLBJ-1 was cultured in PDB medium for 8 days. NRPS: non-ribosomal peptide synthetase; PKS: polyketide synthase; DMATS: dimethylallyl tryptophan synthase; TS: terpene synthase.
