# Supplementary material for: Biosynthesis of Antibiotic Leucinostatins in Bio-control Fungus Purpureocillium lilacinum and Their Inhibition on Phytophthora Revealed by Genome Mining
Source: PLoS Pathog. 2016 Jul 14;12(7):e1005685. doi: 10.1371/journal.ppat.1005685 (PMC4946873; doi:10.1371/journal.ppat.1005685)
Supplement: S12 Table — (DOCX) [file ppat.1005685.s026.docx]

**Table S12 A-domain selectivity of LcsA predicted by NRPSpredictor2.**

|  | Amino acid in leucinostatin A | 10 aa code | NRPSpredictor2 |
| --- | --- | --- | --- |
| A1 | MePro | DVFYVMAAAK | Pro |
| A2 | AHyMeOA | DAIGIGGVIK | Vol |
| A3 | HyLeu | DAILVGAIVK | Leu |
| A4 | AIB | DLGFLAGLFK | Ala |
| A5 | Leu | DASLVGAVLK | Tyr |
| A6 | Leu | DALLVGAVAK | Phe |
| A7 | AIB | DLGFLAGVFK | Ala |
| A8 | AIB | EISI--A-FK | Tcl |
| A9 | β-Ala | DVAMVVAMFK | Gly |
| A10 | DPD | DVGMVGGIYK | Gln |

MePro, 4-methyl-L-proline

AHyMeOA, 2-amino-6-hydroxy-4-methyl-8-oxodecanoic acid

HyLeu, hydroxyleucine

AIB, α-aminoisobutyric acid

DPD, N1, N1-dimethylpropane- 1, 2-diamine
