# Supplementary material for: Biosynthesis of Antibiotic Leucinostatins in Bio-control Fungus Purpureocillium lilacinum and Their Inhibition on Phytophthora Revealed by Genome Mining
Source: PLoS Pathog. 2016 Jul 14;12(7):e1005685. doi: 10.1371/journal.ppat.1005685 (PMC4946873; doi:10.1371/journal.ppat.1005685)
Supplement: S13 Table — (DOCX) [file ppat.1005685.s027.docx]

**Table S13 Primer sequences used in this study.**

| Target gene | Primer type | Sequence | Usage |
| --- | --- | --- | --- |
| lcsA | upF | tccccgcgggga  CTCCTTCGTCCTCTACTGC | constructing KO vector |
|  | upR | aaggaaaaaagcggccgcaaaaggaaaa  GCAAGGATAGCGATTCAGG | constructing KO vector |
|  | downF | cgggatcccgCCTCGTCCCAATTTGCTTT | constructing KO vector |
|  | downR | ggaattccGTCGGCTCATCAGTCCCTA | constructing KO vector |
|  | targetF | ATAAGTTGCCGATATTGAGG | lcsA mutant screening |
|  | targetR | TGGTCTTGGGAGGAGTAGA | lcsA mutant screening |
|  | upcheckF | ACGACGGATGCCACGATAC | all deletion mutant screening |
|  | neoupR | TCATCCTACATAAATAGACGC | All deletion mutant screening |
|  | neodownF | GGTTGAGTTGGTGACGGAT | lcsA mutant screening |
|  | downcheckR | AGTACCCAATTCGCCCTAT | lcsA mutant screening |
|  | qF | CGACTCGGACTCGGTTCAG | qRT-PCR |
|  | qR | TGCCGTGATGGTAGACAAGAG | qRT-PCR |
| lcsC | upF | aattggagctccaccgcgg  CCGATCTAACATGGTCCTC | constructing KO vector |
|  | upR | ttctctagagcggccgc  ATTGTCTATCTGCGGGTGC | constructing KO vector |
|  | downF | ggactagtccTCTTGAAGGCGACAGAGCA | constructing KO vector |
|  | downR | tcccccgggggaACAAACCAGAGGCAACAGC | constructing KO vector |
|  | targetF | GCCATCGTTGGGATAGGTC | lcsC mutant screening |
|  | targetR | TTGAGCGTTTTAGCGGAGC | lcsC mutant screening |
|  | upcheckF | TGCTTTCACAAATACTGGAC | lcsC mutant screening |
|  | downcheckR | GGGAGCATAAAAGGGACAG | lcsC mutant screening |
|  | qF | CCCAGATGCCCTCACTTTT | qRT-PCR |
|  | qR | GACCACCGACTTGAACCAA | qRT-PCR |
| lcsD | upF | aattggagctccaccgcgg  CGTGGATGCTTTCCGTTAC | constructing KO vector |
|  | upR | ttctctagagcggccgc  CTATGCGCCTTCGTTCTGT | constructing KO vector |
|  | downF | tagaactagtggatcc  GCTACTGTTGGCGAAAGAC | constructing KO vector |
|  | downR | atcagttatcgaattc  GGGAATGCCTCACAAATAA | constructing KO vector |
|  | targetF | GTCATCGGGCACTGGAATA | lcsD mutant screening |
|  | targetR | TGCTTGATAAGGCTGTCGC | lcsD mutant screening |
|  | upcheckF | CCATTGGAAGACCAGACAT | lcsD mutant screening |
|  | downcheckR | AAGTGGCTCTGGCTATTTG | lcsD mutant screening |
|  | qF | GCCAAAGTGAGAAGGGACA | qRT-PCR |
|  | qR | CCGCTGGATGAAGTATGAG | qRT-PCR |
| lcsE | upF | cgagctcgGACCTCCCTGTCTGTCCTG | constructing KO vector |
|  | upR | aaggaaaaaagcggccgcaaaaggaaaa  TGTTTGTCCTACTGCCACC | constructing KO vector |
|  | downF | tagaactagtggatcc  AGCCAATAATCGCTCATCT | constructing KO vector |
|  | downR | atcagttatcgaattc  AATAAACTTGTGGGCAACC | constructing KO vector |
|  | targetF | GCGACACGCTGAGAAGGAG | lcsE mutant screening |
|  | targetR | GACCGACCGCAACATAACC | lcsE mutant screening |
|  | upcheckF | ATGGGGAACTGACAAGACC | lcsE mutant screening |
|  | downcheckR | CCACAAACGCTTTAGAAGG | lcsE mutant screening |
|  | qF | TACAGACGCCACCATCAGG | qRT-PCR |
|  | qR | TCAACACGACACGGCTTTC | qRT-PCR |
| lcsF | F | aagcatcgataagctt  CCTCATCTTGCTTACATGTATG | constructing OE vector |
|  | R | cgggctgcaggaattc  CCTTTACAAACAGACATCCA | constructing OE vector |
|  | OE*lcsF*F | TCAGATCAGCCCACTTGTA | OE mutant screening |
|  | OE*lcsF*R | CCCGAAAATGAAAATAGAC | OE mutant screening |
|  | qF | CACAAAGAGGAGGGTTCGG | qRT-PCR |
|  | qR | CTCGCCATGTTCGTGCTAG | qRT-PCR |
| lcsG | qF | GGCTCGTGTCCTCGTCCTG | qRT-PCR |
|  | qR | TCGTCGCCTGCTTGGTATG | qRT-PCR |
| lcsH | qF | ATGAGGCAACCACAACACT | qRT-PCR |
|  | qR | AGACGACCAGCATCCAAGA | qRT-PCR |
| lcsI | qF | GGTCCATCCTTCAAGCAGA | qRT-PCR |
|  | qR | AAGCACGAACAGTTCCACATA | qRT-PCR |
| lcsJ | qF | GTCAAGGGACGACCAGCAC | qRT-PCR |
|  | qR | CTCACGAACCAGGAACCAA | qRT-PCR |
| lcsK | qF | CCCTGCCGCCGTGGCTGTAT | qRT-PCR |
|  | qR | TCGCCCGCTTCGTGTCCGT | qRT-PCR |
| lcsL | qF | TGGAAATGACGGATGGGGATG | qRT-PCR |
|  | qR | GAGGCGAAGCCAGGAAAGG | qRT-PCR |
| lcsM | qF | GAAACCGTCTTTCCGCCTTTA | qRT-PCR |
|  | qR | AGCGAGCCGTACCGTGATG | qRT-PCR |
| lcsB | qF | GCACGAAGCAATCACCTCT | qRT-PCR |
|  | qR | AATCCCACCTCACCCATAA | qRT-PCR |
| lcsN | qF | ACACGAAGCCGTTTACCTA | qRT-PCR |
|  | qR | GAAGACGATCATCATTTGC | qRT-PCR |
| lcsO | qF | GAGATGACGGTCAGGGCAAAG | qRT-PCR |
|  | qR | CGCTCCACCAGAACGCAAG | qRT-PCR |
| lcsP | qF | TAGGCTGGACCGTTGAGAA | qRT-PCR |
|  | qR | GCGCTTTGGTAGAGGAATT | qRT-PCR |
| lcsQ | qF | ACTCGGCGTATGTCAACTT | qRT-PCR |
|  | qR | CAATTCCTCTTCACTCCAAA | qRT-PCR |
| lcsR | qF | CGCATCTGGAATCACCGAC | qRT-PCR |
|  | qR | CGAGGCAACTGACCACGAC | qRT-PCR |
| lcsS | qF | CGATGGGTGCGAGTCTAAG | qRT-PCR |
|  | qR | GTGGGAGGTAACCGATGAA | qRT-PCR |
| lcsT | qF | CCGAGCAAGCGTTTGGAGA | qRT-PCR |
|  | qR | TCAATAAGTGCGGGGCGTA | qRT-PCR |
| VFPBJ_02517 | qF | GCAGGCTTTGCGACATTCC | qRT-PCR |
|  | qR | TCCTCGGGCGAAGATGGTT | qRT-PCR |
| VFPBJ_02518 | qF | CAACAAGCACGACCGAAGC | qRT-PCR |
|  | qR | GCCAAAGACCGTCACTCAG | qRT-PCR |
| VFPBJ_02519 | qF | CTCATCTTCACGTCGTATCTT | qRT-PCR |
|  | qR | GTCCTCGTAGTAGCCCTTG | qRT-PCR |
| VFPBJ_02520 | qF | AAGCCTCGGAAAATACCCC | qRT-PCR |
|  | qR | ACGCTGCTAGTTGTTGACTGC | qRT-PCR |
| VFPBJ_02540 | qF | TTCCCCATCAAGTGGTTCG | qRT-PCR |
|  | qR | GATGCTCAAGGCGTCCAAG | qRT-PCR |
| VFPBJ_02541 | qF | CGGCAAGGCATTCATTCAC | qRT-PCR |
|  | qR | CAGGATTGTGCCCGTTTCA | qRT-PCR |
| VFPBJ_02542 | qF | CAAGGTCCAGGGCTCGCAGAT | qRT-PCR |
|  | qR | CAGCGGGGCAGGGAAACGT | qRT-PCR |
| VFPBJ_02543 | qF | ACTATTTGCCTGCTGTTTG | qRT-PCR |
|  | qR | ATATCGTTGTGCCTCGTAC | qRT-PCR |
| Actin | qF | GCCCTCTGTCCTGGGTCTT | House keeping gene |
|  | qR | ACAGGGAGGCGAGAATGGA | House keeping gene |

KO: knock out; OE: overexpression
