# Supplementary material for: Biosynthesis of Antibiotic Leucinostatins in Bio-control Fungus Purpureocillium lilacinum and Their Inhibition on Phytophthora Revealed by Genome Mining
Source: PLoS Pathog. 2016 Jul 14;12(7):e1005685. doi: 10.1371/journal.ppat.1005685 (PMC4946873; doi:10.1371/journal.ppat.1005685)
Supplement: S14 Table — (DOCX) [file ppat.1005685.s028.docx]

**Table S14 Fungal strains used in phylogemomic analysis.**

| Species | Family | References/Accession number |
| --- | --- | --- |
| *P. lilacinum* PLBJ-1 | Ophiocordycipitaceae | LSBH00000000 |
| *P. lilacinum* PLFJ-1 | Ophiocordycipitaceae | LSBI00000000 |
| *Hirsutella minnesotensis* | Ophiocordycipitaceae | [[1](#_ENREF_1)] |
| *Ophiocordyceps sinensis* | Ophiocordycipitaceae | [[2](#_ENREF_2)] |
| *Ophiocordyceps unilateralis* | Ophiocordycipitaceae | [[3](#_ENREF_3)] |
| *Tolypocladium inflatum* | Ophiocordycipitaceae | [[4](#_ENREF_4)] |
| *Tolypocladium ophioglossoides* | Ophiocordycipitaceae | [[5](#_ENREF_5)] |
| *Beauveria bassiana* | Cordycipitaceae | [[6](#_ENREF_6)] |
| *Cordyceps militaris* | Cordycipitaceae | [[7](#_ENREF_7)] |
| *Claviceps purpurea* | Clavicipitaceae | [[8](#_ENREF_8)] |
| *Metarhizium acridum* | Clavicipitaceae | [[9](#_ENREF_9)] |
| *Metarhizium album* | Clavicipitaceae | [[10](#_ENREF_10)] |
| *Metarhizium anisopliae* | Clavicipitaceae | [[9](#_ENREF_9)] |
| *Metarhizium brunneum* | Clavicipitaceae | [[10](#_ENREF_10)] |
| *Metarhizium guizhouense* | Clavicipitaceae | [[10](#_ENREF_10)] |
| *Metarhizium majus* | Clavicipitaceae | [[10](#_ENREF_10)] |
| *Metarhizium robertsii* | Clavicipitaceae | [[9](#_ENREF_9)] |
| *Pochonia chlamydosporium* strain 123 | Clavicipitaceae | [[11](#_ENREF_11)] |
| *Pochonia chlamydosporium* strain 170 | Clavicipitaceae | LSBJ00000000 |
| *Torrubiella hemipterigena* | Clavicipitaceae | [[12](#_ENREF_12)] |
| *Trichoderma atroviride* | Hypocreaceae | [[13](#_ENREF_13)] |
| *Trichoderma harzianum* | Hypocreaceae | [[14](#_ENREF_14)] |
| *Trichoderma virens* | Hypocreaceae | [[13](#_ENREF_13)] |
| *Trichoderma reesei* | Hypocreaceae | [[15](#_ENREF_15)] |
| *Fusarium avenaceum* | Nectriaceae | [[16](#_ENREF_16)] |
| *Fusarium fujikuroi* | Nectriaceae | [[17](#_ENREF_17)] |
| *Fusarium pseudograminearum* | Nectriaceae | [[18](#_ENREF_18)] |
| *Fusarium verticillioides* | Nectriaceae | [[19](#_ENREF_19)] |
| *Fusarium oxysporum* | Nectriaceae | [[19](#_ENREF_19)] |
| *Fusarium graminearum* | Nectriaceae | [[20](#_ENREF_20)] |
| *Nectria haematococca* | Nectriaceae | [[21](#_ENREF_21)] |
| *Fusarium fujikuroi* | Nectriaceae | [[17](#_ENREF_17)] |
| *Stachybotrys chartarum* | Stachybotriaceae | [[22](#_ENREF_22)] |
| *Ustilaginoidea virens* | Hypocreales incertae sedis | [[23](#_ENREF_23)] |
| *Acremonium chrysogenum* | Hypocreales incertae sedis | [[24](#_ENREF_24)] |
| *Arthrobotrys oligospora* | Orbiliaceae | [[25](#_ENREF_25)] |
| *Monacrosporium haptotylum* | Orbiliaceae | [[26](#_ENREF_26)] |
| *Saccharomyces cerevisiae* | [Saccharomycetaceae](http://www.ncbi.nlm.nih.gov/Taxonomy/Browser/wwwtax.cgi?mode=Undef&id=4893&lvl=3&keep=1&srchmode=1&unlock) | [[27](#_ENREF_27)] |

1. Lai Y, Liu K, Zhang X, Zhang X, Li K, Wang N, et al. Comparative genomics and transcriptomics analyses reveal divergent lifestyle features of nematode endoparasitic fungus *Hirsutella minnesotensis*. Genome Biol Evol. 2014; 6(11): 3077-3093. doi: 10.1093/gbe/evu241

2. Hu X, Zhang Y, Xiao G, Zheng P, Xia Y, Zhang X, et al. Genome survey uncovers the secrets of sex and lifestyle in caterpillar fungus. Chinese Sci Bul. 2013; 58(23): 2846-2854. doi: 10.1007/s11434-013-5929-5

3. De Bekker C, Ohm RA, Loreto RG, Sebastian A, Albert I, Merrow M, et al. Gene expression during zombie ant biting behavior reflects the complexity underlying fungal parasitic behavioral manipulation. BMC Genomics. 2015; 16(1): 620. doi: 10.1186/s12864-015-1812-x

4. Bushley KE, Raja R, Jaiswal P, Cumbie JS, Nonogaki M, Boyd AE, et al. The genome of *Tolypocladium inflatum*: evolution, organization, and expression of the cyclosporin biosynthetic gene cluster. PLoS Genet. 2013 Jun; 9(6): e1003496. doi:10.1371/journal.pgen.1003496

5. Quandt CA, Bushley KE, Spatafora JW. The genome of the truffle-parasite *Tolypocladium ophioglossoides* and the evolution of antifungal peptaibiotics. BMC Genomics. 2015; 16(1): 553. doi: 10.1186/s12864-015-1777-9

6. Xiao G, Ying SH, Zheng P, Wang ZL, Zhang S, Xie XQ, et al. Genomic perspectives on the evolution of fungal entomopathogenicity in *Beauveria bassiana*. Sci Rep. 2012; 2. doi: 10.1038/srep00483

7. Zheng P, Xia Y, Xiao G, Xiong C, Hu X, Zhang S, et al. Genome sequence of the insect pathogenic fungus *Cordyceps militaris*, a valued traditional Chinese medicine. Genome Biol. 2011; 12(11): R116. doi: 10.1186/gb-2011-12-11-r116

8. Schardl CL, Young CA, Hesse U, Amyotte SG, Andreeva K, Calie PJ, et al. Plant-symbiotic fungi as chemical engineers: multi-genome analysis of the Clavicipitaceae reveals dynamics of alkaloid loci. PLoS Genet. 2013; 9(2): e1003323. doi:10.1371/journal.pgen.1003323

9. Gao Q, Jin K, Ying S-H, Zhang Y, Xiao G, Shang Y, et al. Genome sequencing and comparative transcriptomics of the model entomopathogenic fungi *Metarhizium anisopliae* and *M. acridum*. PLoS Genet. 2011; 7(1): e1001264. doi:10.1371/journal.pgen.1001264

10. Hu X, Xiao G, Zheng P, Shang Y, Su Y, Zhang X, et al. Trajectory and genomic determinants of fungal-pathogen speciation and host adaptation. P Natl Acad Sci. 2014; 111(47): 16796-16801. doi: 10.1073/pnas.1412662111

11. Larriba E, Jaime MDLA, Carbonell-Caballero J, Conesa A, Dopazo J, Nislow C, et al. Sequencing and functional analysis of the genome of a nematode egg-parasitic fungus, *Pochonia chlamydosporia*. Fungal Genet and Biol. 2014; 65: 69-80. [doi:10.1016/j.fgb.2014.02.002](http://dx.doi.org/10.1016/j.fgb.2014.02.002)

12. Horn F, Habel A, Scharf DH, Dworschak J, Brakhage AA, Guthke R, et al. Draft genome sequence and gene annotation of the entomopathogenic fungus *Verticillium hemipterigenum*. Genome Announc. 2015; 3(1): e01439-01414. doi: 10.1128/genomeA.01439-14

13. Kubicek CP, Herrera-Estrella A, Seidl-Seiboth V, Martinez DA, Druzhinina IS, Thon M, et al. Comparative genome sequence analysis underscores mycoparasitism as the ancestral life style of Trichoderma. Genome Biol. 2011; 12(4): R40. doi: 10.1186/gb-2011-12-4-r40

14. Baroncelli R, Piaggeschi G, Fiorini L, Bertolini E, Zapparata A, Pè ME, et al. Draft whole-genome sequence of the biocontrol agent *Trichoderma harzianum* T6776. Genome Announc. 2015; 3(3): e00647-00615. doi: 10.1128/genomeA.00647-15

15. Martinez D, Berka RM, Henrissat B, Saloheimo M, Arvas M, Baker SE, et al. Genome sequencing and analysis of the biomass-degrading fungus *Trichoderma reesei* (syn. *Hypocrea jecorina*). Nat Biotechnol. 2008; 26(5): 553-560. doi: 10.1038/nbt1403

16. Lysøe E, Harris LJ, Walkowiak S, Subramaniam R, Divon HH, Riiser ES, et al. The Genome of the Generalist Plant Pathogen *Fusarium avenaceum* Is Enriched with Genes Involved in Redox, Signaling and Secondary Metabolism. PLoS One. 2014; 9(11): e112703. doi: 10.1371/journal.pone.0112703

17. Wiemann P, Sieber C, Von Bargen KW, Studt L, Niehaus E-M, Espino JJ, et al. Deciphering the cryptic genome: genome-wide analyses of the rice pathogen *Fusarium fujikuroi* reveal complex regulation of secondary metabolism and novel metabolites. PLoS Pathog. 2013; 9(6): e1003475. doi:10.1371/journal.ppat.1003475.g001

18. Gardiner DM, McDonald MC, Covarelli L, Solomon PS, Rusu AG, Marshall M, et al. Comparative pathogenomics reveals horizontally acquired novel virulence genes in fungi infecting cereal hosts. PLoS Pathog. 2012; 8(9): e1002952. doi: 10.1371/journal.ppat.1002952

19. Ma L-J, Van Der Does HC, Borkovich KA, Coleman JJ, Daboussi M-J, Di Pietro A, et al. Comparative genomics reveals mobile pathogenicity chromosomes in *Fusarium*. Nature. 2010; 464(7287): 367-373. doi: 10.1038/nature08850

20. Cuomo CA, Güldener U, Xu J-R, Trail F, Turgeon BG, Di Pietro A, et al. The *Fusarium graminearum* genome reveals a link between localized polymorphism and pathogen specialization. Science. 2007; 317(5843): 1400-1402. doi: 10.1126/science.1143708

21. Coleman JJ, Rounsley SD, Rodriguez-Carres M, Kuo A, Wasmann CC, Grimwood J, et al. The genome of *Nectria haematococca*: contribution of supernumerary chromosomes to gene expansion. PLoS Genet. 2009; 5(8): e1000618. doi: 10.1371/journal.pgen.1000618

22. Semeiks J, Borek D, Otwinowski Z, Grishin NV. Comparative genome sequencing reveals chemotype-specific gene clusters in the toxigenic black mold Stachybotrys. BMC Genomics. 2014; 15(1): 590. doi: 10.1186/1471-2164-15-590

23. Zhang Y, Zhang K, Fang A, Han Y, Yang J, Xue M, et al. Specific adaptation of *Ustilaginoidea virens* in occupying host florets revealed by comparative and functional genomics. Nat Commun. 2014; 5:3849. doi: 10.1038/ncomms4849

24. Terfehr D, Dahlmann TA, Specht T, Zadra I, Kürnsteiner H, Kück U. Genome Sequence and Annotation of *Acremonium chrysogenum*, Producer of the β-Lactam Antibiotic Cephalosporin C. Genome Announc. 2014; 2(5): e00948-00914. doi:10.1128/genomeA.00948-14

25. Yang J, Wang L, Ji X, Feng Y, Li X, Zou C, et al. Genomic and proteomic analyses of the fungus *Arthrobotrys oligospora* provide insights into nematode-trap formation. PLoS Pathog. 2011; 7(9): e1002179. doi:10.1371/journal.ppat.1002179

26. Meerupati T, Andersson K-M, Friman E, Kumar D, Tunlid A, Ahrén D. Genomic mechanisms accounting for the adaptation to parasitism in nematode-trapping fungi. PLoS Genet. 2013; 9(11): e1003909. doi:10.1371/journal.pgen.1003909

27. Goffeau A, Barrell B, Bussey H, Davis R, Dujon B, Feldmann H, et al. Life with 6000 genes. Science. 1996; 274(5287): 546-567. doi: 10.1126/science.274.5287.546
